# Electrocatalysts for Inorganic and Organic Waste Nitrogen Conversion

**DOI:** 10.1021/acscatal.4c01398

**Published:** 2024-06-14

**Authors:** Danae A. Chipoco Haro, Luisa Barrera, Haldrian Iriawan, Antonia Herzog, Nianhan Tian, Andrew J. Medford, Yang Shao-Horn, Faisal M. Alamgir, Marta C. Hatzell

**Affiliations:** † School of Materials Science and Engineering, Georgia Institute of Technology, North Avenue 771 Ferst Dr., Atlanta, Georgia 30332, United States; ‡ George W. Woodruff School of Mechanical Engineering, Georgia Institute of Technology, 770 Ferst Ave, Atlanta, Georgia 30309, United States; ¶ Department of Materials Science and Engineering, Massachusetts Institute of Technology, 77 Massachusetts Avenue, Cambridge, Massachusetts 02139, United States; § Research Laboratory of Electronics, Massachusetts Institute of Technology, 77 Massachusetts Avenue, Cambridge, Massachusetts 02139, United States; ∥ School of Chemical & Biomolecular Engineering, Georgia Institute of Technology, Atlanta, Georgia 30332, United States; ▽ Department of Mechanical Engineering, Massachusetts Institute of Technology, 77 Massachusetts Avenue, Cambridge, Massachusetts 02139, United States

**Keywords:** organic nitrogen, electrocatalytic waste nitrogen conversion, real waste matrixes, colorimetric methods, electrocatalyst design, inorganic nitrogen

## Abstract

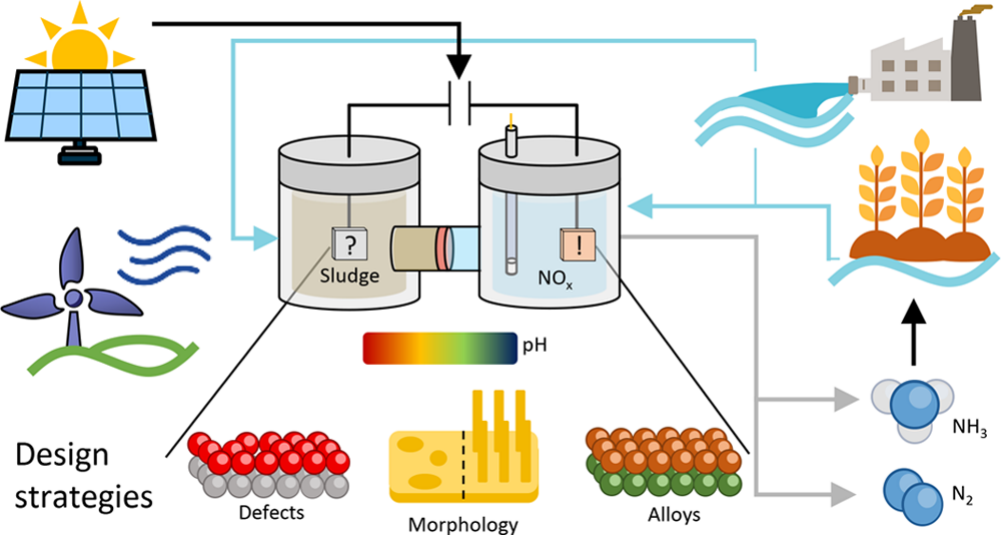

Anthropogenic activities have disrupted the natural nitrogen cycle, increasing the level of nitrogen contaminants in water. Nitrogen contaminants are harmful to humans and the environment. This motivates research on advanced and decarbonized treatment technologies that are capable of removing or valorizing nitrogen waste found in water. In this context, the electrocatalytic conversion of inorganic- and organic-based nitrogen compounds has emerged as an important approach that is capable of upconverting waste nitrogen into valuable compounds. This approach differs from state-of-the-art wastewater treatment, which primarily converts inorganic nitrogen to dinitrogen, and organic nitrogen is sent to landfills. Here, we review recent efforts related to electrocatalytic conversion of inorganic- and organic-based nitrogen waste. Specifically, we detail the role that electrocatalyst design (alloys, defects, morphology, and faceting) plays in the promotion of high-activity and high-selectivity electrocatalysts. We also discuss the impact of wastewater constituents. Finally, we discuss the critical product analyses required to ensure that the reported performance is accurate.

## Introduction

The application of nitrogen fertilizers is currently disrupting the nitrogen cycle. As the world population continues to increase, the use of ammonia (NH_3_) fertilizer is expected to grow rapidly.^1^ Current industrial-scale NH_3_ production is achieved through the Haber–Bosch process, with 80% of the output by volume going toward the manufacturing of modern synthetic fertilizers.^2,3^ The Haber–Bosch process is a thermocatalytic process that reduces nitrogen (N_2_) with hydrogen (H_2_) to produce NH_3_.^4^ The Haber–Bosch process, although important for sustaining the global population, has some disadvantages: it uses H_2_ derived from fossil fuels, as a feedstock, and its operation requires high temperatures (250 to 400 °C) and pressures (100 to 300 bar).^5−10^ These conditions result in high energy consumption (540 to 800 kJ/mol_NH3_).^4^ As the Haber–Bosch process is powered by natural gas and coal, these high energy demands result in the annual production of 1.2% of global greenhouse gas emissions.^8^

**Figure 1:**
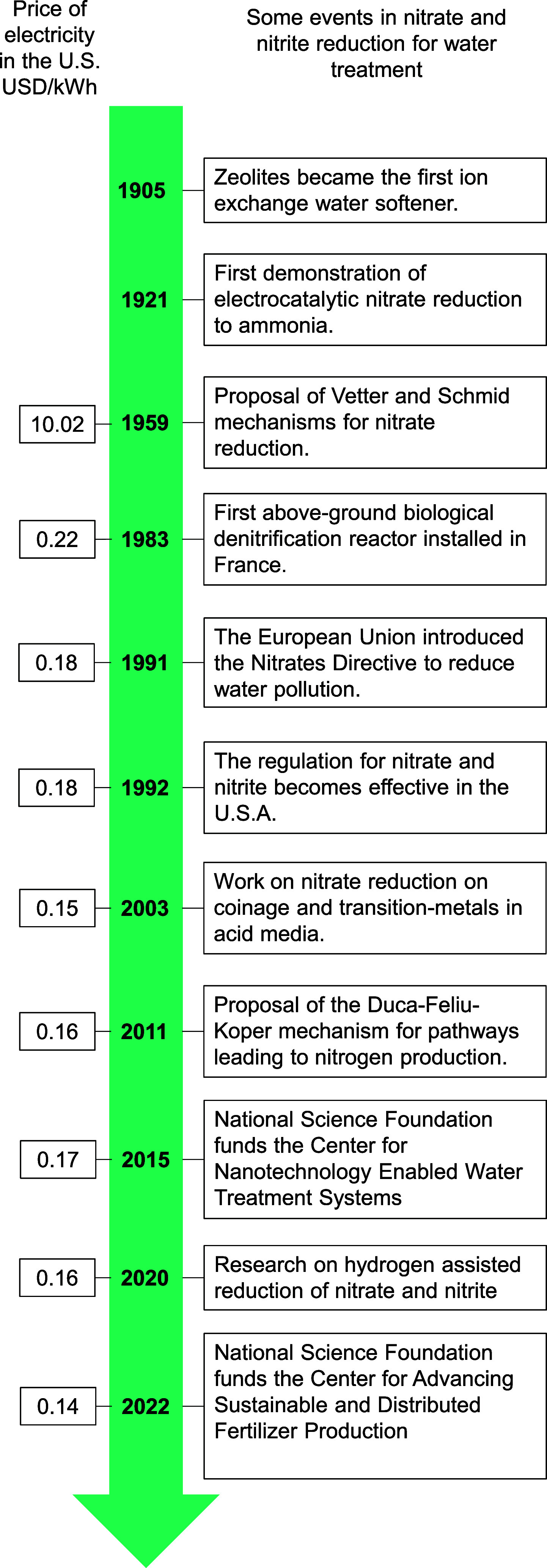
Relevant events for NO_3_^–^ and NO_2_^–^ reduction research^16−24^ alongside the average price of electricity to ultimate customers in the U.S.^25,26^ Prices are corrected for inflation to April 2024 using the Inflation Calculator of the U.S. Bureau of Labor Statistics.^27^

The increase in fertilizer use has resulted in not only an increase in greenhouse gas emissions but also an increase in toxic oxyanions (e.g., nitrate and nitrite) in groundwater and wastewater. This increase in organic and inorganic nitrogen waste can trigger eutrophication of water bodies.^11,228^ Specifically, the increased concentration of nitrate (NO_3_^–^) in surface and groundwater is harmful to the environment and human health.^12^ The US Environmental Protection Agency (EPA) regulates the maximum allowed concentration of NO_3_^–^ in drinking water (10 mg/L). Some of the health risks associated with NO_3_^–^-containing water intake include methemoglobinemia, cancer, and birth defects.^13^ Nitrite (NO_2_^–^) is 10 times more toxic than NO_3_^–^ for humans.^14,15^ The remediation of NO_3_^–^ and NO_2_^–^ is a growing concern around the world^16−24^ (Figure 1^25−27^) due to the dispersed nature of water-based contaminants, which requires a range of technologies capable of operating with different volumes, flow rates, and feedwater compositions.

There has also been an increase in organic nitrogenous compounds in wastewater, mainly contained within manure and sludge. Common components in the influent waste stream of a wastewater treatment facility are urea, amino acids, pesticides, pharmaceuticals, and dyes.^28,29^ Organic nitrogen contamination can also originate from the wastewater treatment process itself, where microbial treatment processes contribute approximately 50% of the total dissolved organic nitrogen in wastewater treatment plant effluents.^28^ The presence of organic nitrogen contamination is expected to grow due to the combined effects of increased population growth and increased access to water treatment.^30^ When released in the water cycle, organic nitrogen also promotes eutrophication, as organic nitrogen can be used as both a nutrient and an energy resource by microbial communities. Organic nitrogen can transform into toxic nitrogenous disinfection byproducts that are harmful to human and animal health.^28,31^

Here, we discuss progress made in the design of an electrocatalyst that can transform NO_3_^–^, NO_2_^–^, and organic nitrogen. We aim to provide an overview of the reaction mechanism and identify the primary products formed by each reaction. We also discuss the role that other water-based constituents play during electrocatalysis. Finally, we examine standard procedures for product analysis in the field. We hope that the discussion and parallelism between electrochemical NO_3_^–^ and NO_2_^–^ and organic nitrogen oxidation support the scaling of the electrochemical nitrogen conversion of real nitrogen-containing wastewater.

## State of the Art for Waste Nitrogen Treatment

The main treatment of NO_3_^–^ and NO_2_^–^ occurs through biological denitrification, where NO_3_^–^ is converted to N_2_. Biological denitrification is selective in reducing the concentration of NO_3_^–^ to N_2_. This treatment does not produce additional waste streams or demineralization. The organisms responsible for the biological denitrification processes are heterotrophic or autothrophic. Heterotrophic bacteria require a carbon source to obtain energy for cellular activity, among which methanol (CH_3_OH) is the most common. In the respiration step, NO_3_^–^ is reduced to N_2_ while CH_3_OH is oxidized to water and carbon dioxide (CO_2_) (eq 1). Autotrophic organisms oxidize organic matter to produce energy and release electrons. One of the drawbacks of biological denitrification is that denitrification can lead to nitrous oxide pollution, as a byproduct of some enzymatic processes.^32^ Another drawback of biological denitrification is the long startup time (∼25 to >100 days).^33−36^
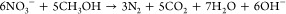
1

Distributed and decentralized treatment facilities use emerging technologies, such as treatment facilities, ion-exchange resins, membranes, and catalytic reduction methods. Ion exchange resin systems remove ions (e.g., NO_3_^–^) from wastewater through the exchange of NO_3_^–^ with receptor sites (e.g., typically chloride).^37^ Sodium chloride (NaCl) or sodium bicarbonate (NaHCO_3_) regenerates resins, allowing NO_3_^–^ to be concentrated in a smaller volume.^38^ The main challenges for resin-based technologies are associated with high operational cost (e.g., waste disposal and energy consumption) and high capital cost associated with materials limitations (e.g., ion exchange capacity and durability). Since ion exchange methods remove NO_3_^–^ from water, resin-based treatment technologies produce a brine with high concentrations of NO_3_^–^ (1000 mg/L) and other salts. Biological methods cannot remove NO_3_^–^ from the brine due to harsh conditions. Therefore, the cost estimates for the disposal of the waste brine are $0.02 to $0.36 per 1000 gal. or $0.005 to $0.095 per 1000 L.^39,40^ The energy for the disposal of waste associated with the pretreatment and chemical processes is 2.3 to 8.9 kWh/m^3^ feed.^41−43^ Finally, the adsorption processes are limited by the adsorption capacity of a given resin. The adsorption capacity of a resin depends on the resin composition and functional groups on the resin surface, as well as the wastewater composition, among others.^44^ Today, most modern anion exchange resins can only achieve adsorption capacities of the order of 5.0 mg-NO_3_^–^/g-resin^45^ to 277.77 mg-NO_3_^–^/g-resin,^46^ with most on the order of 15 mg-NO_3_^–^/g-resin.^20,44^ Resins need frequent regeneration and decay over time depending on the resin and operating conditions.^47^ Supercritical water tests at 380 °C for 1h showed a degradation based on total organic carbon in the range of 93% and 96%.^48^ Some resins are regenerated, but require a longer contact time compared to single-use resins.^49−51^

Catalytic reduction of NO_3_^–^ for wastewater treatment is selective and does not require post-treatment of the waste. In this method, NO_3_^–^ is catalytically reduced through three processes on bimetallic catalysts (Figure S1): (i) reduction of NO_3_^–^ to NO_2_^–^ and further reduction to NH_3_ or N_2_,^52,53^ (ii) regeneration of the catalyst,^53,54^ and (iii) pH neutralization using buffering agents.^55^ This process requires a metal promoter for the reduction of NO_3_^–^, a noble metal for the regeneration of the catalyst,^52^ and a reducing agent (e.g., H_2_ or formic acid).^56^ One of the drawbacks of catalytic reduction processes is the short durability of the catalyst (5 cycles, 200 h).^33,57^ Catalysts can be deactivated by the formation of a passivation layer^57^ or by agglomeration of catalysts.^34,58^

Similarly to NO_3_^–^ and NO_2_^–^ treatment processes, the main removal approach of organic-N utilizes biological treatment.^28^ Autotrophic organisms can take up the low-molecular-weight organic-N directly to form NH_3_. Heterotrophic organisms hydrolyze high-molecular-weight organic-N, like proteins and amino acids, into smaller molecules such as NH_3_ and volatile fatty acids (VFA). In addition to the drawbacks previously mentioned, (e.g., the lack of nutrient recovery and the long start-up time), biological treatment processes can be limited in the treatment of organic-N by the presence of nonbiodegradable or toxic species.^59−61^ Emerging alternative technologies currently pursued in the literature include coagulation, electrocoagulation, and electrochemical oxidation approaches.^28,31,59−62^ Coagulation introduces coagulant species (e.g., metallic, polymeric) to promote the formation of colloids that can agglomerate into flocs for easier removal.^31^ The success of the coagulation processes thus inherently results in the formation of a secondary waste sludge. Optimization of the process depends on the solution conditions (e.g., pH and conductivity), the coagulant addition, and the organic-N species treated. Electrocoagulation introduces the coagulants through a sacrificial electrode and can benefit from side-reactions destabilizing the pollutants.^31,63^ However, electrocoagulation intrinsically depends on frequent electrode replacement, which can lead to high operational costs, and generates a secondary waste sludge as the result of the coagulation process.^31,59,63^ Electrochemical oxidation processes are promising due to the versatility of the approach, the possible treatment of large volumes of contaminants, and the potentially high degree of organic matter degradation while avoiding the generation of secondary waste streams.^60,61^ The growing body of research has applied direct and indirect anodic oxidation as well as Fenton-based approaches to treat synthetic solutions containing phenols, dyes, pesticides, drugs, as well as real industrial effluents.^60,61,64^

Among catalytic processes, electrochemical catalytic reduction and oxidation processes stand out because catalytic processes can be easily coupled with renewable energy technologies, such as photovoltaics and wind turbines, thereby decreasing the carbon footprint. Another advantage is that no reducing or oxidizing agent is needed, as the electric potential can directly reduce nitrate or oxidize organic nitrogen. There is also the potential for tuning selectivity on the basis of applied potential, in addition to catalyst design.

## Electrochemical Reduction of Nitrate and Nitrite

**Figure 2:**
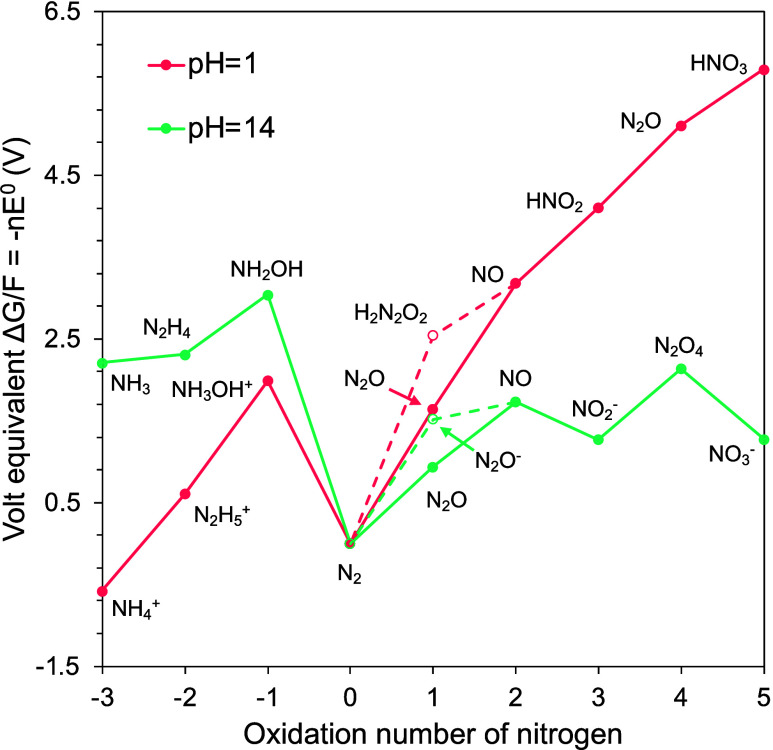
Frost diagram of N-species at pH 1 and 14.

Nitrogen can be found with oxidation states that range from −III to +V. As shown in the Frost diagram (Figure 2), the thermodynamic stability of a N compound with different oxidation states depends on the average Gibbs molar energy and the specific conditions of the solution.^65^ A compound located at an energy lower than that of the connecting line between neighboring species is more stable than neighboring N compounds under a certain pH. Thus, the neighboring compounds will tend to be comproportionate to the most stable lower-energy compound. In contrast, compounds located at an energy higher than that of the connecting line between neighboring species are less stable than those of neighboring species. Thus, the less stable species will tend to be disproportionate (e.g., H_2_N_2_O_2_ and N_2_O^–^). The Frost diagram (Figure 2) is also a useful graphical representation of the standard potentials associated with N-compound transformations, where the standard Gibbs energy of the nitrogen transformation compound in N_2_ is equivalent to the standard potential times the oxidation state of N. The standard potential between the transformations of two compounds can be obtained by the slope connecting the two species (e.g., difference in the volt equivalents divided by difference in the oxidation states). The diagram shows that ammonium (NH_4_^+^) and N_2_ are the most thermodynamically stable products under acidic conditions, where an alkaline solution can promote the stability of oxidized N-compounds (oxidation state >0).

Electrochemical nitrate reduction (NO_3_RR) and nitrite reduction (NO_2_RR) can produce a number of nitrogen compounds, including NO_2_^–^, nitric oxide (NO), nitrous oxide (N_2_O), N_2_, and NH_4_^+^ or NH_3_.^66^ However, the two predominant final products are N_2_ and NH_3_, which are also the most thermodynamically stable products.^67^ NO_3_^–^ is reduced to N_2_ through a 5-electron process (eq 2) and to NH_3_ through an 8-electron process (eq 3). NO_2_^–^ is reduced to N_2_ through a 3-electron process (eq 4)^68^ and NH_4_^+^ through a 6-electron process (eq 5).
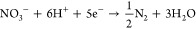
2
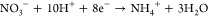
3
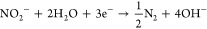
4
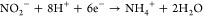
5The reaction pathway depends on the concentration of the reactant, the electrolyte, the applied potential, and the catalyst.^69,70^ This account highlights progress made toward understanding the catalytic reaction mechanisms.

### Electrocatalytic Nitrate and Nitrite Reaction Mechanisms

**Figure 3:**
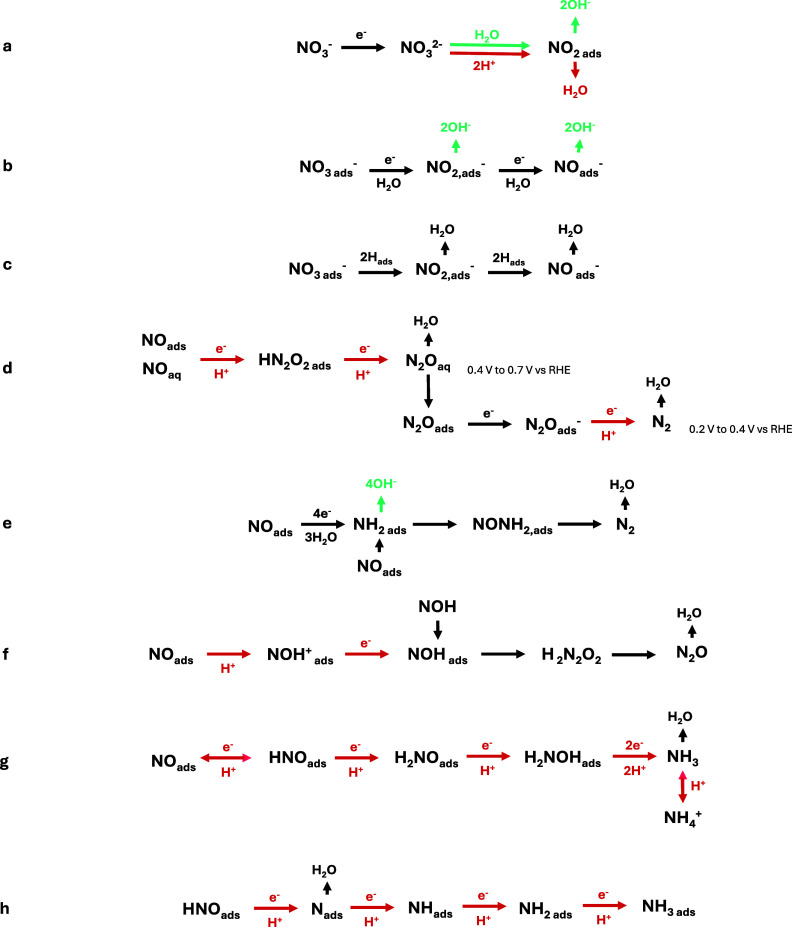
Reaction mechanisms of electrochemical NO_3_^–^ and NO_2_^–^ reduction. (a) Indirect autocatalytic pathway.^73,74^ (b, c) Direct reduction reaction mechanisms (b) without and (c) with H. (d) Vooys–Koper–Chumanov mechanism.^79,80^ (e) Duca–Feliu–Koper mechanism.^81−83^ (f) Nitric oxide to N_2_O mechanism.^84−86,89^ (g) Mechanisms on Pt electrode.^87,88^ (h) Mechanisms on Fe single atoms.^93^ Alkaline media reactions are in green; acidic media reactions are in red.

The reduction of NO_3_^–^ to NO_2_^–^ is the rate-determining step (RDS) in the reduction of NO_3_^–^ to N_2_ or NH_3_.^71^ The NO_3_^–^ reduction may follow (i) a direct electrocatalytic or (ii) an indirect autocatalytic reduction pathway. The indirect autocatalytic pathway occurs mainly in acidic media, but can sometimes also occur in alkaline media.^72^ In the indirect autocatalytic pathway, NO_3_^–^ reacts with a solvated electron. In the presence of a solvated electron, NO_3_^–^ produces the dianion radical NO_3_^2–^. NO_3_^2–^ reacts with water to generate the resulting NO_2_^–^. The presence of multivalent cations or ionizing radiation may generate the solvated electron (Figure 3a, green for alkaline media, and red for acid media).^73,74^

In acidic media, the concentration of NO_2_ may be reduced through an autocatalytic reduction pathway. First, NO_2,ads_ protonates (NO_2,ads_ + e^–^ → NO_2_^–^, NO_2_^–^ + H^+^ → HNO_2_). Then, the Schmid mechanism proceeds with reduction to NO (HNO_2_ + H^+^ + e^–^ → NO + H_2_O). Under acidic conditions, NO_3_^–^ exists either protonated or as an anion (H^+^ + NO_3_^–^ ↔ HNO_3_), and it can now undergo several different reaction pathways. The protonated NO_3_^–^ can react with NO to form HNO_2_ (2NO + HNO_3_ + H_2_O → 3HNO_2_).^75^ The Vetter mechanism between HNO_3_ and HNO_2_ has both NO_2_ and N_2_O_4_ as products (HNO_3_ + HNO_2_ → N_2_O_4_ + H_2_O), which are in equilibrium (N_2_O_4_ ↔ 2NO_2_).^18^ For acidic conditions, HNO_2_ dissociation is prevalent due to the presence of HNO_3_.^76^ At high (>4 M) concentrations of HNO_3_, NO (2HNO_3_ ⇌ 2NO + O_2_ + H_2_O) and N_2_O_4_ (2HNO_3_ ⇌ N_2_O_4_ + O_2_ + H_2_O) are formed because of a shift in the equilibrium.^77^ In alkaline media, the autocatalytic pathway proposed proceeds through a reaction between adsorbed NO_3_^–^ and NO_2_^–^ and H adsorbed on the surface of the catalyst. It is assumed that this pathway is the limiting stage of the process.^78^

In the direct reduction reaction mechanism, NO_3_^–^ is directly reduced by an electrochemically generated electron at an electrode surface (Figure 3b). NO_3_^–^ and NO_2_^–^ can also be directly reduced by H_ads_ (Figure 3c).

Transition metal electrodes (Pt, Ru, Ir, Rh, Pd, and Au) follow a Vooys–Koper–Chumanov mechanism for the reduction of NO to N_2_ and N_2_O. At high potentials (ca. 0.4 to 0.7 V vs RHE), N_2_O was observed as the main product. The proposed mechanism involves adsorbed and aqueous NO (Figure 3d). At lower potentials (ca. 0.2 to 0.4 V vs RHE), N_2_ was observed as the main product.^79^ N_2_ is formed by the further reduction of N_2_O (Figure 3d).^80^

NO_ads_ can be further reduced through the Duca–Feliu–Koper mechanism to N_2_ (Figure 3e). This mechanism was first observed on Pt(100) surfaces and in alkaline media. Pt(100) surfaces stabilize the NO_ads_ to recombine with NH_2,ads_ species and form NONH_2,ads_. The unstable intermediate NONH_2,ads_ decomposes into N_2_.^81,82^ Nitramide (NH_2_NO) was also experimentally identified as an intermediate in the reduction to N_2_.^83^

Nitric oxide can also be protonated to NOH (Figure 3f).^84,85^ The dimerization of NOH yields hypo-nitrous acid H_2_N_2_O_2_. H_2_N_2_O_2_ decomposes into N_2_O, which is an intermediate for N_2_ formation.^86^

Ammonia is another product of the NO_2_^–^ reduction. An electrochemical–electrochemical mechanism was observed, instead of an electrochemical–chemical mechanism, on Pt electrodes (Figure 3g).^87^ Nitric oxide can be protonated to azanone (HNO).^88^ Azanone can be further protonated to aminooxidanide (H_2_NO) through the RDS and further reduced to H_2_NOH.^89^ NH_3_ is in equilibrium with the ammonium cation (NH_4_^+^). Density functional theory (DFT) calculations showed that the N–N formation is not feasible for Pt(211). Geometry optimization does not yield a stable configuration of *cis*-(NO-NO)_ads_ on the Pt(211) surface. In addition, the formation of *trans*-(NO-NO)_ads_ requires a high kinetic barrier (Δ*G*_a_ = 1.34 eV).^90^ Therefore, the Pt(211) surface shows a dominant formation of NH_3_ rather than N–N bond formation.

Similarly, Pd(100) favors the NOH_ads_ formation, which then dissociates to N_ads_ through a water-mediated proton shuttle (eq 6). N_ads_ is the intermediate for N_2_O formation, which then leads to N_2_ formation. On the other hand, Cu(100) stabilizes the N–H bond through HNO_ads_. This favors the formation of ammonium over N_2_.^91^
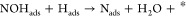
6

The reaction pathway was also dependent on the coverage of the surface. DFT calculations on Cu(211) demonstrated that at 1/6 monolayers of coverage, NOH_ads_ was the favorable intermediate, while at 1/2 monolayers of coverage, HNO_ads_ was the favorable intermediate toward NH_3_.^90^ Aminooxidanide can also be hydrogenated in the presence of hydrogen radicals (eqs 7 and 8). Electrochemical nitrate reduction experiments at room temperature and DFT calculations in strained Ru clusters support this reaction mechanism.^92^
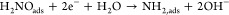
7
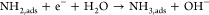
8

DFT calculations on Fe single metal atoms (Fe SAC)^93^ suggest another hydrogenation pathway through adsorbed N_2_. The calculated minimum energy pathway shows NO_ads_ as a key intermediate. Subsequent reduction of NO_ads_ to HNO_ads_ and HNO_ads_ to N_ads_ could also be potential limiting steps. N–N coupling is energetically unfavorable, thus the hydrogenation steps (Figure 3h) are favored.^93^

### Electrocatalysts for Nitrate Reduction

**Figure 4:**
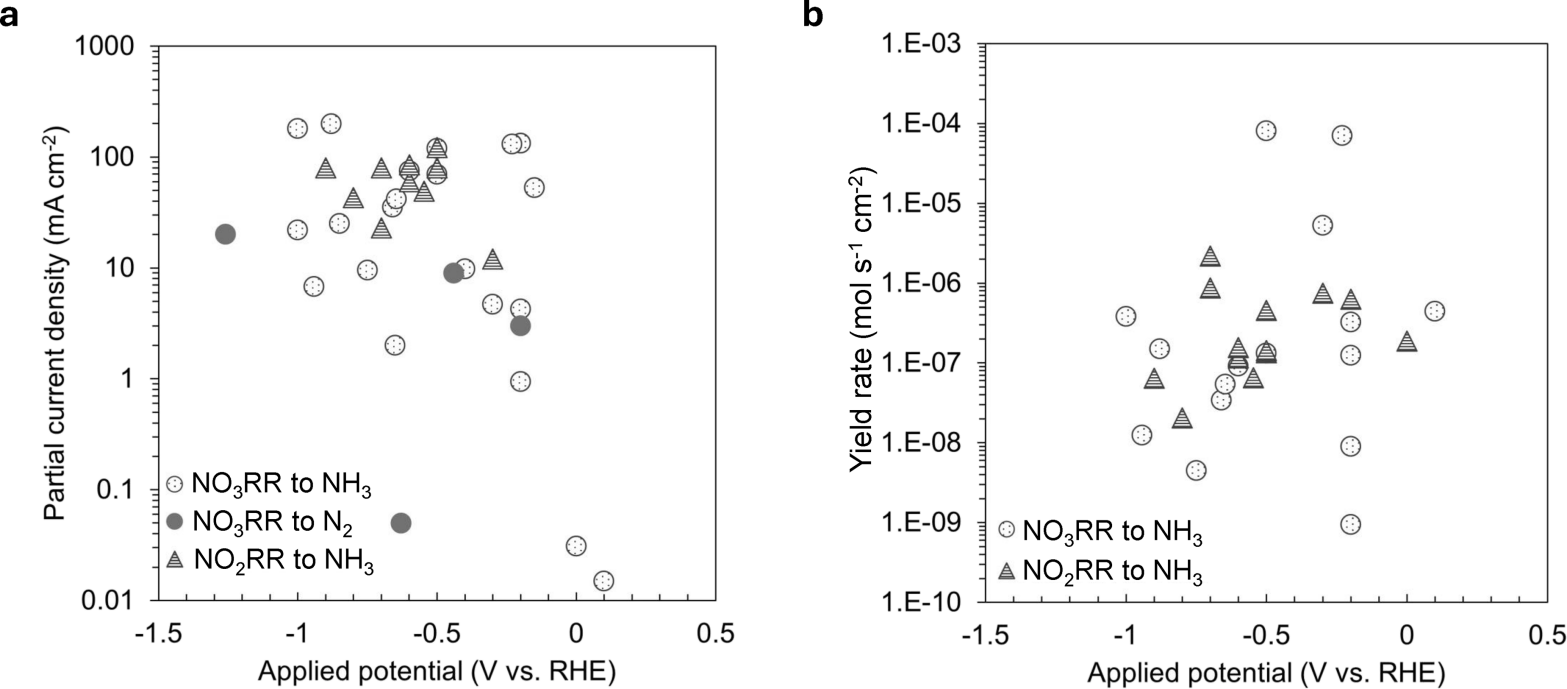
Literature review of (a) partial current density and (b) yield rate of NO_3_RR and NO_2_RR. Details on the reference of each catalyst can be found in Figure S6 and Tables S1 through S4.

The choice of an electrocatalyst plays a crucial role in guiding the selectivity, activity, and durability of the electrochemical conversion of nitrate and nitrite. In the sections that follow, we examine various electrocatalysts that have been studied and have been classified into three main types: noble metals, non-noble metals, and carbon-based catalysts (Figure 4).

#### Noble Metal Electrocatalysts

Ruthenium supported on carbon exhibits pseudo-first-order rate kinetics for the reduction of NO_3_^–^, as shown by tests in acidic medium (pH 5.5) where NO_2_^–^ was not detected as a solution-phase (intermediate) species. This suggests that the NO_2_RR is faster than nitrate to nitrite reduction (RDS). Furthermore, DFT calculations showed a strong adsorption of NO_2_^–^ and NO in Ru clusters.^95^ Such computational insights could be supported by in situ spectroscopy techniques, such as surface-enhanced Raman spectroscopy and surface-enhanced infrared spectroscopy, where vibrational modes associated with N=O bending or stretching from both solution-phase and adsorbed NO_2_^–^ or NO_3_^–^ species were detected in other material systems.^96−98^ A ruthenium core–shell structure synthesized with O-doped Ru expands the Ru unit cell, generating tensile strain. The tensile strain decreases the bond strength of H–H, thus suppressing the hydrogen evolution reaction (HER) and favoring adsorbed hydrogen atom (H_ads_).^92^ DFT-simulated Ru doped on the (101) orientation of TiO_2_ has also been shown to facilitate the adsorption of NO_2_^–^ by accelerating charge transfer on the TiO_2_ electrode surface.^99^ The applied potential and the adsorption strengths of the intermediates have a large impact on the selectivity and activity of the catalyst.^79^ DFT calculations of the NO_3_RR with Rh indicated scaling relations between O and N and the NO_3_RR intermediates. By using DFT and in situ X-ray absorption near edge structure (XANES) and extended X-ray absorption fine structure (EXAFS), it was found that strong adsorption of O and N resulted in more favorable N_2_ formation at all potentials (e.g., Fe and Co), while moderate adsorption of O and N resulted in more selective formation of NH_3_ or NO. Rh exhibited the highest predicted electrocatalytic activities for NH_3_ production at positive potentials (0–0.4 V), followed by Cu, Pt, and Pd.

Alloying may increase the activity of a catalyst.^100^ The concentration of different metals usually follows a volcano relationship, i.e., as the concentration of the alloyed material increases, the performance increases until an “optimum” point, after which increasing the concentration decreases the activity of the catalyst to ammonia. In the case of PtRu alloys, the highest current density (activity) was achieved at 22% Ru. Lower (10%) or higher (37% and 52%) Ru loading showed lower activity than the 22% Ru content. All the alloys showed higher activity to NH_3_ than the Pt counterpart (0% Ru). This can be attributed to the stronger binding of NO_3_^–^ to the catalyst surface. The higher d-band center of Ru compared to that of Pt strengthens the adsorption of NO_3_^–^. This leads to greater coverage of NO_3_^–^ on the catalyst surface, helping to reduce NO_3_^–^.^101^ A small amount of In in Pt (0.25 wt %) increases the NO_2_^–^ conversion. NO_2_RR occurs in Pt sites, so increasing In content (>1 wt %) decreases the activity of the catalyst. The role of In in the catalyst is to change the electronic structure of Pt to prevent hydrogenation toward NH_4_^+^ and favor the combination of nitrogen intermediates (N_2_ selectivity of 94%).^102^

When considering a catalyst, it is also important to consider the pH in which it is going to be operating since this can change the reaction pathways, thus creating potential poisoning at the surface. PtRu supported on carbon showed an increase in catalytic activity with an increase in pH from 0 to 3 and a plateau to pH 7. The plateau may be due to effects from the reaction environment or changes in the catalyst as a result of pH. Interestingly, faradaic efficiency toward NH_3_ increased with pH, going from 54% at pH 1 to 93% at pH 7.^103^ Overall, as pH increases, the reaction kinetics stops being dependent on H^+^, where the hydrogen source is provided by H_2_O. Alloys can also tune the selectivity of the catalyst. Pt has great selectivity for the production of NH_3_ in acidic media (only product detected). When alloyed with Sn, the alloy showed an enhanced reduction of NO_3_^–^ to NO_2_^−^, and a change in selectivity toward H_2_NOH (82% selectivity).^104^ NO_2_RR on Rh is affected by NO_ads_ poisoning. NO_ads_ is also affected by pH due to the dissociation and adsorption of HNO_2_ while the dissociation of NO_2_^–^ is pH-independent. Surface-enhanced Raman spectroscopy shows an increase in the NO_ads_ coverage on the Rh surface at a lower pH. Increasing pH slows the rate at which NO_ads_ is deposited on the surface. In alkaline media, the predominant species is NO_2_^–^ rather than HNO_2_. The hydrogenation steps are influenced by the NO/H coverage ratio (as shown in Pd-based catalysts). This ratio depends on the pH of the system due to the relative abundance of protonated and deprotonated NO_2_.^76^

The single AuCu(111) atom with surface Cu vacancies (CuV) shows higher catalytic activity and selectivity toward NH_3_ than the Cu catalyst and CuV catalyst counterparts. X-ray photoelectron spectroscopy (XPS) shows charge transfer from Cu to Au atoms. The alloy lowers the energy barrier for water dissociation, and the CuVs increase the *H binding ability compared to the Cu nanosheets catalyst. Thus, the AuCu(111) single atom with surface CuV catalyst inhibits HER but favors the hydrogenation steps for NH_3_ formation.^105^ Ag was introduced in a controlled way into NiO nanosheets to increase the yield for NH_3_ to over 1000 μg h^–1^ cm^–2^. Ag film sputtered on NiO nanosheet arrays on a C cloth creates a Ag nanoarray (Ag@NiO/CC). NO_2_RR DFT on Ag(111) and Ag(100) showed a preferable pathway NO_2,ads_ → NO_2_H_ads_ → NO_ads_ → NOH_ads_ → N_ads_ → NH_ads_ → NH_2,ads_ → NH_3,ads_. In this case, the potential-determining step (PDS) on Ag(111) is the hydrogenation of NO, while the PDS on Ag(100) is the hydrogenation of NO_2_. The DFT calculations show that Ag is highly active for NO_2_^–^ to NH_3_, which explained the increase in NH_3_ yield after adding Ag to NiO.^106^

Palladium shows selectivity for NH_3_ production in the NO_3_RR. Pd does not promote the formation of N_2_O, which is a key intermediate for N_2_ production in alkaline media. Pd has a good affinity for hydrogen, which helps in the selectivity of NH_3_. Different Pd facets showed different contributions to NO_3_^–^ reduction. Pd(111) reduces NO_3_^–^ to NO_2_^–^, while the Pd(100) facet reduces NO_2_^–^ to NH_3_.^107^ Pd–Cu alloys are more selective for N_2_ than NH_3_.^108^ In addition, Pd with Ag create of sites that are more favorable for binding N than the surface sites present in either Pd or Ag. DFT calculations show that as the Ag ratio increases, the N-binding energy becomes weaker. In the triatomic ensemble that is formed on the catalyst, Pd-rich sites have more favorable energy compared to Pd–Ag sites or pure Pd(111). Thus, alloying small amounts of Ag improves the reactivity of the Pd catalyst (Pd_95_Ag_5_ nanoparticles (NPs)).^109^ Alloying Pd with Ir shows an increased selectivity of NO_2_RR toward NH_3_. The theoretical maximum H-coverage of Ir(111) is higher than that of Pd(111), which favors NH_3_ production. The larger orbitals of 5d metals compared to 4d metals lead to stronger interactions with H.^110^

#### Non-noble Metal-Based Catalysts

Noble-metal-based catalysts have shown good catalytic activity and selectivity. However, noble metals are expensive and scarce, which limits noble metal applications. In this context, non-noble-metal-based electrocatalysts are a good alternative. The products of the NO_3_RR on Cu(111) and Cu(100) are NO_2_^–^ and hydroxylamine (NH_2_OH). Thermodynamic studies on Cu(100) show that these products are more favorable than NH_3_ formation, while kinetic analysis has shown the preferable formation of NH_3_ over NH_2_OH. This could be due to an additional potential dependence, such that at low overpotentials, NH_2_OH was produced, and with increasing overpotentials, NH_3_ was more favorable.^111^ In acidic media, Cu(111) and Cu(100) NO_3_RR produce NH_4_^+^, according to thermodynamic studies.^112^ Kinetic studies showed that NH_3_ formation (OHN_ads_ → N_ads_, kinetic barrier = 0.13 eV) is more favorable than NH_2_OH (HON_ads_ → H_2_ON_ads_, kinetic barrier = 0.34 eV).^111^ In alkaline media, NO_3_^–^ reduces to NO_2_^–^ at lower potentials on Cu(111) than on Cu(100), but Cu(100) reduced NO_2_ to NH_2_OH at a higher rate than Cu(111). Also, Cu(111) deactivates more easily than Cu(100) in both alkaline and acidic media due to the higher activity of HER on the surface of Cu(111).^112^ Furthermore, the pH level also affects the competitive relationship of NO_3_RR with HER. Calculations of Gibbs free energy showed that in acidic environments (pH < 5.63), Cu(111) would thermodynamically favor HER over NO_3_^–^ reduction to NH_4_^+^, but Cu(100) would favor NO_3_^–^ reduction to NH_4_^+^ over HER. In alkaline media, Cu(111) surfaces would favor NO_3_^–^ reduction to NH_3_.^113^ In neutral media, Cu/Cu_2_O nanowire arrays (NWAs) show a higher performance toward NH_3_ production compared to CuO NWAs counterparts. The higher performance is attributed to electron transfer at the Cu/Cu_2_O interface suppressing HER formation and facilitating NOH_ads_ formation as supported by DFT calculations.^114^

Doping is a catalyst design strategy that allows electronic structure tuning of the material to optimize the energy needed for intermediate adsorption. Doping can also reduce the competing reaction of HER.^115,116^ In the NO_2_RR, DFT shows that the adsorption of NO_2_^–^ on Cu_3_P is stronger than that of hydrogen. The Cu_3_P(202) surface has the lower uphill energy for the PDS of NO hydrogenation.^116^ Cu single atom (SA) sites in N-doped carbon enhance the catalytic activity of NO_3_RR toward NH_3_ because Cu_1_–N_4_ coordination suppresses HER. The N-doped carbon dual-mesoporous structure improves mass transfer kinetics, which aids in NO_3_RR. DFT calculations show that NH_3_ production is thermodynamically favorable in Cu_1_–N_4_ coordination when compared to Cu clusters, which show N_2_ production.^117^ Combining Ni with Cu results in an upshift of the Cu d-band center toward the Fermi level, resulting in better NO_3_^–^ adsorption.^118^ However, introducing an excess of Ni (≥30:70, Cu:Ni ratio) can lower the activity and selectivity of the catalyst, as enhanced adsorption can result in surface poisoning, and Ni has a lower selectivity than Cu. DFT calculations show that at high ratios of Ni on Cu, the reaction free energy of NH_2_ increases, leading to a decrease in selectivity toward NH_3_.^119^ Similarly, Fe doping on Cu catalysts showed an upward shift of the 3d-band of Cu, resulting in better catalytic performance for NO_3_RR.^120^ The creation of nanointerfaces in alloys through nanodecoration can alter the selectivity of catalysts toward a specific product. Cu foam was decorated with Pt nanoparticles, such that H_2_ adsorbs onto Pt in a dissociative way, creating H_ads_ for hydrogenation steps in reduction of NO_3_^–^. In addition, Pt catalyzes hydrogenation reactions.^121^ Ni foam increased the catalytic activity of Cu for NO_3_RR to NH_3_ when compared with Cu foam as a support. Ni adsorbs hydrogen strongly, thus facilitating the generation of H_ads_ and enhancing NH_3_ production.^122^

Alloying some metals can also be detrimental to selectivity toward NH_4_^+^. Iridium nanoparticles showed good selectivity of NO_2_RR toward NH_4_^+^ at pH 6.4 (near 100%). This good selectivity was related, among other factors, to the H coverage on Ir surfaces. However, when alloyed with Cu, NH_4_^+^ molecules tend to overbound at the Ir-atop sites. As a result, Cu–Ir activity toward NH_4_^+^ decreases.

The introduction of oxygen vacancies (OVs) in CuO nanoparticles through plasma increases the energy of NO_3_^–^ adsorption (−0.93 to −0.5 eV), but more than 1 OV decreased the energy of adsorption to more negative values (−1.84 eV for 2 OVs and −2.08 eV for 3 OVs). In the case of HER, 1 OV leads to a decrease in the energy barrier (from 0.41 to 0.04 eV). Introducing more OVs decreases the free energy to negative values (−0.6 eV for 2 OVs and −0.68 for 3 OVs). The greater formation of OVs, along with a decrease in surface crystallinity and lower electron density, increases NH_3_ formation, but decreases ammonia selectivity due to an increase in HER.^123^ Cu nanoribbons^124^ facilitate NO_3_RR yet hinder HER. The free energy of adsorption of NO_3_^–^ on Cu(100) is −0.127 eV. The free energy of adsorption of NO_3_^–^ is lower when atomic defects are present on Cu(100), at −0.502 eV. By upshifting the d-band center of Cu, the interaction between Cu(100) and defects produced a remarkable NH_3_ yield of 650 mmol g_cat_^–1^ h^–1^ at −0.15 V (vs RHE) and a Faraday efficiency of 95.3%.

Iron carbide (Fe_3_C) has high electrical conductivity and showed good catalytic activity for NO_3_RR and selectivity toward NH_3_. An appropriate ratio of Fe^3+^/Fe^2+^ and the d-band centers is crucial for the strong adsorption of reactants, intermediates, or products. A higher ratio of Fe^3+^ results in larger empty d-orbitals. The Fe^3+^/Fe^2+^ ratio and the d-band centers showed a volcano-like relationship with respect to the NO_3_RR. The DFT calculation in FeN_2_O_2_ showed an increase in the Fermi level compared to the FeN_4_ configuration. As a result, NO_3,ads_ is more likely to form. The energy barrier for N_ads_, an important intermediate in the reaction, is lowered.^125^ The faradaic efficiency of FeN_4_ toward ammonia in alkaline (pH 13) and neutral media is similar, but with an improvement in the overpotentials. The tests in acidic media (pH = 1) showed a decrease in activity and selectivity.^93^

Titanium has been studied as a catalyst because of its stability and ability to suppress HER.^126^ In neutral media, TiO_2–*x*_ showed an increase in the catalytic activity when compared to that of TiO_2_ counterparts. Theoretical calculations on TiO_2_(101) showed that the introduction of OVs caused the position of the Fermi level to move into the conduction band. Furthermore, the vacancies were filled with oxygen, which weakened the O–N bond.^127^ A hollow structure of Ti was studied under Ar flow through the hollow structure.^128^ The Ar flow optimizes the mass transport, which improves the current density and yield. There is a decrease in faradaic efficiency toward NH_3_ and an increase in faradaic efficiency toward NO_2_^–^ and NH_2_–OH. The study proposes an increase in local pH, which can interfere with NH_3_ production. Metal phosphides have a partial positive charge on the metal atom and a partial negative charge on the phosphorus atom. The charge transfer effect can readily and reversibly generate adsorbed H. The alternate adsorption of NO_*x*_ and H on the metal and phosphorus sites prevents competition for active sites. At the same time, H is delivered to the NO_*x*_ adsorbed species. DFT confirmed charge transfer and H adsorption in the Ni_2_P catalysts. The proposed mechanism suggested the formation of Ni_2_P–H, where NO_2_^–^ was reacted to form the NO intermediate. Subsequently, NO was hydrogenated to H_2_NO, H_2_NOH, and NH_3_.^129^ Ni_2_P nanosheet arrays showed good catalytic activity due to the 3D architecture, which helped expose active sites and promote the diffusion of reactants. DFT calculations showed that Ni_2_P(111) showed a lower energy barrier for hydrogenation steps compared to formation of N_2_O_2_, which explained the selectivity toward NH_3_ over N_2_.^130^ CoP(112) showed a higher catalytic activity than Co(OH)F. DFT showed that the energy for NO desorption required more energy than that for NO hydrogenation. This resulted in good faradaic efficiency for NO_2_RR to NH_3_ (FE = 90% ± 2%).^131^

Studies on the effect of pH on Ti foil showed that acidic conditions (0.77 and 2.95 pH) favor NH_3_ formation. Ti forms a hydride under moderately acidic conditions. This could reduce the faradaic efficiency toward NH_3_ formation, because part of the cathodic current is directed toward the formation of the hydride. The faradaic efficiency of the Ti foil decreased (85% to 50%) after 8 h under acidic conditions. This could be caused by surface poisoning or the effect of hydride formation on selectivity.^132^

Ti plates can also support other catalysts. A Co–P catalyst supported on Ti plates demonstrated high catalytic activity and selectivity toward NH_3_. Co–P catalysts have a good affinity for H adsorption, which helps in hydrogenation reactions.^133^ Oxygen vacancies in TiO_2_ increase titaniums catalytic activity and the faradaic efficiency of NO_2_RR toward NH_3_ production. Oxygen vacancies induce metallic behavior on TiO_2_, which increases the conductivity of the catalyst.^127^ In addition, the adsorption sites were more favorable for NH_3_ production.^134^ Vanadium-doped TiO_2_ nanobelt arrays possess an exposed TiO_2_(101) crystal plane, where V replaces the four coordinated Ti atoms on the TiO_2_(101) surface. X-ray diffraction showed an increased *d*-spacing of V-TiO_2_ due to incorporation of V. Evaluation of the partial density of states showed that V 3d participates in the composition of the valence and conduction bands. Also, it narrows the band gap due to the formation of an energy level between the valence and conduction bands. This results in an increase in electrical conductivity and catalytic activity compared to TiO_2_. The PDS in the NO_2_RR was NO_2,ads_ hydrogenation, as calculated by DFT. Doping with V decreases the energy of the NO_2,ads_ hydrogenation from 0.64 eV (TiO_2_) to 0.53 eV (V-TiO_2_). Moreover, V-TiO_2_ PDS is *NO to NO rather than NO_2,ads_ hydrogenation.^135^ Nonmetallic elements can also be used as dopants. TiO_2_ was doped with P. DFT showed that doping resulted in an increase in conductivity in TiO_2_ by closing the band gap from 2.10 to 0.44 eV. The increase in conductivity results in faster reaction kinetics and facilitates charge transfer on the catalyst’s surface. The differences in the length of Ti–P bonds, when compared to Ti–O, introduced the OVs. Moreover, P acted as a strong adsorption site for NO_2_^–^. The Gibbs free energy calculation for NO_2_^–^ adsorption and other crucial intermediate formation (NO, N, NH, and NH_2_) showed that P-TiO_2_ had a lower Gibbs free energy than TiO_2_. Furthermore, the Gibbs free energy for H adsorption was higher in P-TiO_2_ (0.72 eV) compared to TiO_2_ (0.24 eV). This results in a hindered HER.^136^

Co/CoO nanosheets allow electron transfer from metallic Co to CoO, resulting in electron-deficient Co. Turnover frequencies calculations showed that Co is active for NO_3_RR, but not selective. Co/CoO promotes selectivity toward NH_3_, both experimentally and through theoretical calculations.^137^ Amorphous borides provide a long-range disorder that results in active sites for catalytic reactions. These active sites are active for NO_2_RR to NH_3_ (FE = 95.2%), with low faradaic efficiency for N_2_ (≤2%) or HER (≤5%).^138^

#### Carbon-Based Catalysts

As discussed, metal-based electrocatalysts for the NO_3_RR have been widely studied. However, concerns about the possible release of metal ions in acid environments motivated the study of nonmetal-based catalysts.^139^ In this context, carbon nanomaterials have been used as catalysts for oxygen reduction and HER due to carbons high stability and conductivity properties.^140^

Multiwalled carbon nanotube (MWCNT) catalysts showed better catalytic activity toward NO_3_RR to NH_3_ than oxidized MWCNT (OCNT) and reduced OCNT. This suggests that pristine carbon nanotubes are active for NO_3_RR. Zeta potential measurements showed that OCNT possesses a negative charge on the surface that may repel NO_3_^–^.^140^ The NO_3_^–^ reduction in N-doped graphene demonstrated that pyridinic-N had better catalytic activity for NO_3_RR than pyrrolic-N.^139^ C-doping can increase the catalytic activity as a result of a disturbance in the charge density of carbon, resulting in more free electrons. F-doping on carbon increased the disorder of the material, causing increased catalytic activity toward NH_3_. Furthermore, F-doped carbon suppresses HER and DFT calculations indicate lower energies of NO_ads_ and NOH_ads_ as compared to carbon.^141^

Doping materials facilitate electron transfer as a result of the creation of electron donors. In this sense, the conductivity of a material could be increased. B-doped diamond is a catalyst capable of reducing NO_3_^–^ with high selectivity toward N_2_.^142^ A reduced pretreatment of the B-doped diamond (CR-BDD) resulted in surface hydrogenation (C–H bond formation) that contributes to the hydrophobicity of CR-BDD. The hydrophobicity of the materials weakens the competition of HER with NO_3_RR. However, hydrophobicity might also inhibit NO_3_^–^ adsorption. In addition, C–H bonds give rise to shallow acceptor states that facilitate electron transfer between CR-BDD and NO_3_^–^. The amount of B doping affects the level of hydrogenation. The highest C–H bond ratio was obtained by a B/C ratio of 1%, as at higher B/C ratios, the C–H bond ratio decreased. The highest faradaic efficiency for N_2_ formation (44.5%) and NO_3_RR (85%) was observed at a B/C ratio of 1%. Increased doping with B decreased faradaic efficiency for N_2_ formation.^143^

### Electrocatalytic Reduction of Nitrate in Real Wastewater

In recent years, several works have addressed the treatment of NO_3_^–^ in real water matrices. NO_3_^–^ in wastewater from industries such as chemical production, printing, fly ash cleaning, textiles, as well as contaminated water sources like irrigation and river water, was successfully reduced to NH_3_.^144−150^ Recovery of NH_3_ in large-scale applications remains an outstanding issue. This issue prompted the development of prototypes that integrate the NH_3_ production and recovery. These prototypes are at the bench scale (e.g., electrochemical flow cells^145,148^ and adsorbent materials^151^) and at the pilot scale.^144^

Bi_2_O_3_ nanosheets were grown on carbon cloth to pair the high catalytic activity of Bi with the large surface area and favorability to NO_3_^–^ adsorption found in carbon cloth.^147^ When treating real wastewater, such as fly ash washing wastewater, high concentrations of Cl^–^ are present in the fly ash washing wastewater solution (37.5 g L^–1^ compared to NO_3_^–^ concentrations [NO_3_^–^] = 75.48 mg L^–1^). The presence of Cl^–^ actually improved the conversion of Bi^3+^ to Bi^0^, which then increased the activity of the catalyst, through the presence of highly active sites at the grain boundaries.

Cu-, Co-, and Ni-based materials have been successfully engineered to treat real wastewater compositions with low NO_3_^–^ concentrations (<20 mg L^–1^) through a combination of surface modifications and different form factors. Cu cubes with heterostructured Cu-CuO skin had high selectivity and faradaic efficiency toward NH_3_ formation during the treatment of natural river water ([NO_3_^–^] = 18.12 mg L^–1^).^150^ Using DFT, the improved behavior compared to unmodified Cu cubes was attributed to higher driving forces for subsequent *NO reactions following the NO_3_^–^-to-NO_2_^–^ reduction step, more favorability toward NH_3_ desorption, as well as hindering competing H_2_ reactions. Cu nanorods formed on the Cu wire had high selectivity toward the formation of NH_3_ when treating wastewater from the Stockholm water plant ([NO_3_^–^] = 3.47 mg L^–1^).^151^

Cu single-atom aerogels achieved high selectivity toward NH_3_ production during the treatment of industry wastewaters ([NO_3_^–^-N] = 500 mg L^–1^). In comparison to bulk Cu, DFT calculations indicate that single atoms on carbon aerogels had more favorable NO_3_^–^/NO_2_^–^ adsorption and kinetics toward NH_3_ production.^148^ Finite element simulations highlight improved NO_2_^–^ to NH_4_^+^ reactivity thanks to nanoconfinement effects resulting from using the aerogel support. CuO on Cu foam used in an electrified membrane flow-cell can effectively treat chemical industry wastewaters ([NO_3_^–^-N] = 436 ± 15 mg L^–1^).^145^

A variety of Co-based electrodes prepared on Ti mesh treated real printing wastewater ([NO_3_^–^-N] = 208 mg L^–1^ and [NO_2_^–^-N] = 32 mg L^–1^), with the best behavior reported for CoP.^146^ Electrochemical reduction in the presence of scavengers highlights the dependence of NO_3_RR on the direct electron transfer reaction. DFT indicates that CoP is more thermodynamically favorable toward NO_3_^–^ as opposed to hydrogen adsorption. Contrastingly, Ni foam was “self-activated” by strong adsorption of H* on the electrode surface, resulting in Ni(OH)_2_ formed due to corrosion and chemical oxidation.^144^ The indirect reduction pathway thus promoted the reduction of NO_3_^–^ to NH_3_ in the treated factory workshop wastewater ([NO_3_^–^-N] = 2552 ± 38 mg L^–1^).

## Electrochemical Oxidation of Nitrogen-Containing Organic Compounds

Additional nutrient recovery from complex matrices, such as wastewater sludge and livestock manure, can be achieved by the electrochemical oxidation of organic N compounds. Herein, organic N compounds refer to nitrogen bound within molecules with carbon-hydrogen bonds (e.g. aminoacids) and carbon-oxygen bonds (e.g. urea). While NH_3_ production and recovery remain an emerging focus of the field,^64,152−154^ there has been an abundance of literature examining electrochemical oxidation of organic matter for sludge treatment.^155−157^ Common electrode materials include titanium-based catalysts, platinum, boron-doped diamond (BDD), and carbon. These are widely used to treat organic pollutants, including textile dyes, pharmaceuticals, and pesticides, and have been extensively covered in recent work.^60,64,158,159^ Other materials like Ni and Fe have well-established behaviors for organic oxidation and are currently being explored for real waste treatment.^59,64,156^ The following section covers the possible electrochemical oxidation pathways and examples of catalysts being implemented in real waste matrices.

### Electrocatalytic Organic-Nitrogen Oxidation Reaction Mechanisms

**Figure 5:**
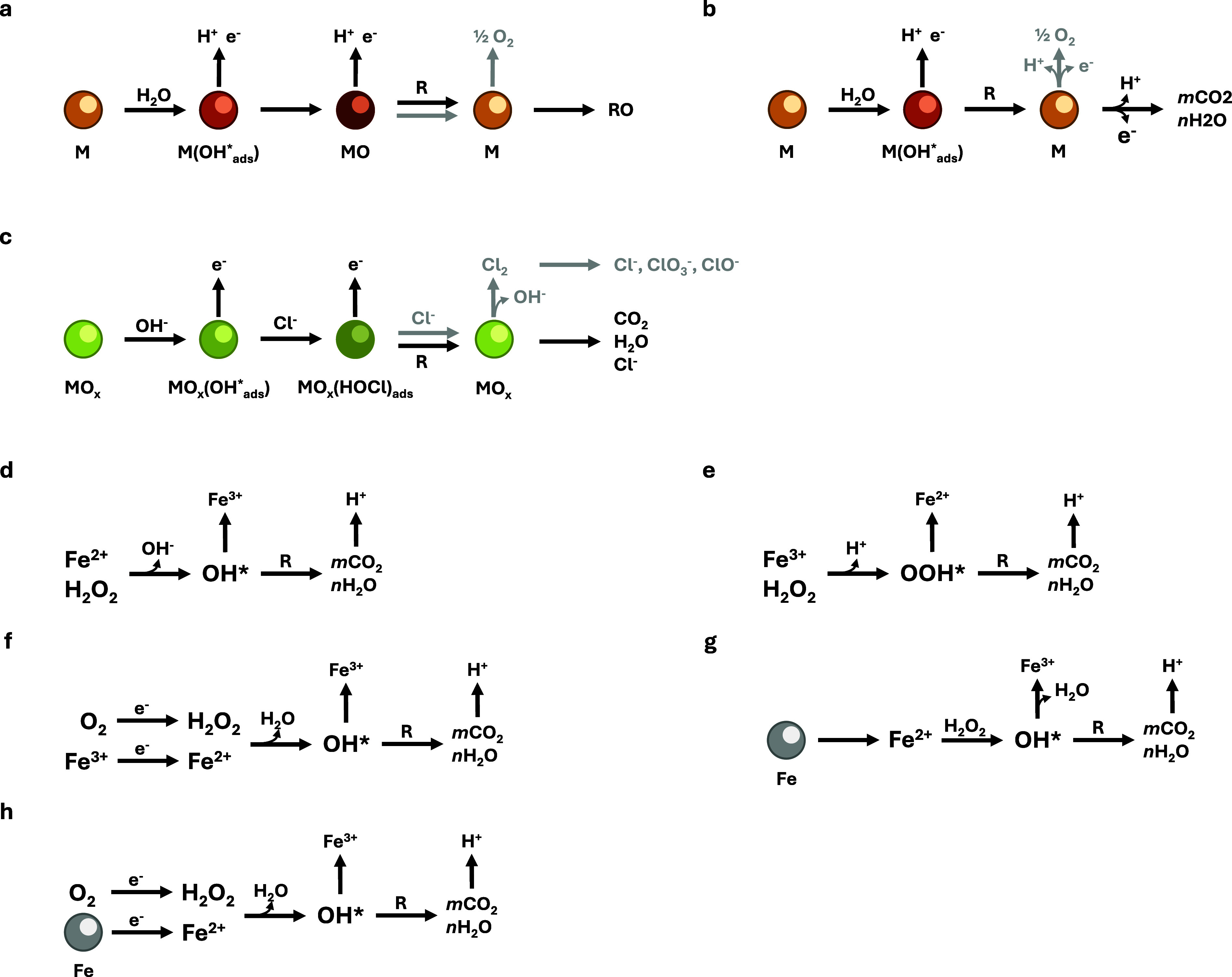
Organic species oxidation mechanisms.^160,163−165^ Direct oxidation mechanism on (a) an active electrode and (b) a nonactive electrode. (c) Indirect oxidation mechanism mediated by active chlorine species. (d)–(h) Fenton-based processes: (d) classic Fenton, (e) Fenton-like, (f) electro-Fenton, (g) electrochemical Fenton (also called Galvano-Fenton), (h) electro-Fenton with sacrificial anode. Gray arrows and species indicate a competing pathway. M: metal surface, R: organic species.

Similarly to electrochemical NO_3_^–^ reduction, electrochemical organic-N oxidation can take place via direct and indirect routes by using reactive radicals. Direct electrochemical oxidation of organic species takes place under an applied potential, following Figure 5a or b. This difference in mechanism highlights the importance of the metal electrode choice. Selective or partial organic oxidation behavior has been reported on “active” metals with low oxygen evolution overpotentials (e.g., IrO_2_). The organic oxidation is possible due to the strong interaction of the physisorbed hydroxyl radical with the electrode surface, resulting in the formation of metal oxides (MO). The presence of MO allows for a direct interaction between chemisorbed organic species and the higher oxidation states of the M electrode and results in selective/partial oxidation pathways being available.^158,160^ Complete electrochemical oxidation of organics to CO_2_ is possible on “non-active” metals with high oxygen evolution overpotentials (e.g., SnO_2_) thanks to the weakly bound physisorbed M(OH)_ads_^*^.^156−158,160^ Indirect electrochemical oxidation of organic species takes place through the electrochemical production of radicals such as OH* and Cl^–^.^59,64,158,161^ OH* participates similarly to M(OH)_ads_^*^ in the complete combustion of organics. Active chlorine species have several forms, as the anode can convert chloride to chlorine, gaseous chlorine, hypochlorous acid, or hypochlorite ions (Figure 5c). These active chlorine species can then participate in the complete combustion of the organic species. Active chlorine is widely used in wastewater treatment approaches due to chlorides presence in many contaminated streams and chlorines effectiveness in degrading organic species.^160,162^ Additional common treatment processes are based on the Fenton approach, where H_2_O_2_ and Fe^2+^ react to form OH*, in the classic process (Figure 5d), or OOH*, in the Fenton-like process (Figure 5e). While an effective approach, the main drawbacks of Fenton-based treatment procedures are the dependence on large volumes of reagents and the formation of an Fe-based sludge. These issues can be minimized by using electrochemical techniques such as the electro-Fenton approach (Figure 5f), where the H_2_O_2_ is produced in situ and the Fe^3+^ is regenerated into Fe^2+^, or the electrochemical Fenton (also called Galvano-Fenton) approach (Figure 5g), where the Fe^2+^ is generated in situ through the use of a sacrificial anode.^163^ An emerging approach, electro-Fenton with sacrificial anode, looks to produce both reagents in situ.^164^ To illustrate the direct and indirect mechanisms, urea is discussed as an example.

Urine is the source of 70% to 80% of nitrogen in municipal wastewater,^166^ and it is composed mainly of water (95%) and urea (2%).^167^ Urea electrooxidation (UEO) using Ni electrocatalyst also undergoes direct (eqs 9 through 11) or indirect oxidation (eqs 12 through 14).^168^ Ni surface is usually oxidized by air, so an oxidized form is usually in contact with the electrolyte. In alkaline media, Ni electrochemically forms Ni(OH)_2_ on the surface of the electrode. The electrochemical reaction (eq 12) forms NiOOH. NiOOH participates in the urea oxidation, yielding several byproducts such as OCN^–^, NH_4_^+^, CO_3_^2–^, and NO_2_^–^. The urea oxidation on Ni is possible due to the reversibility of Ni^3+^ and Ni^2+^.^169^ In this study, no direct urea oxidation was observed, but the urea conversion was around 90%.^170^ Kinetic studies suggest that urea would bind to Ni^3+^ on two sites: (i) via both amine groups or (ii) via amine and ketone groups.^171^ Chlorine-mediated oxidation of urea is possible through the generation of reactive chlorine species that form HCl and other species such as CNO^–^, NO_3_^–^, NO_2_^–^, N_2_, and CO_2_.^172^ Tests with BiO/TiO_2_ show that chloride ions in solution can form gaseous chloride (>2 V vs NHE) or chloride radicals (<2 V vs NHE). Then, urea reacts with chlorate, resulting in CO_2_ and to mono-, di-, or trichloroamine. Finally, these chloroamines undergo redox reactions to yield N_2_, NO_2_^–^, or NO_3_^–^.^173^
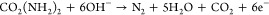
9
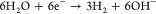
10
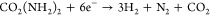
11
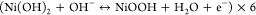
12
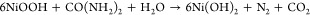
13
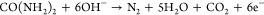
14

Most organic matter oxidation is proposed to be reacted indirectly, resulting in the degradation of organic matter to CO_2_. However, it remains unclear which oxidation process (direct, indirect, or a combination of both) drives the degradation of the organic-N toward NH_3_.^161^ Some have hypothesized that the NH_3_ may be formed from the oxidation of organic-N-containing compounds such as amino acids reacting through a pseudo Fenton-like process (eqs 15 and 16); however, this has yet to be verified.
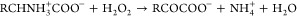
15
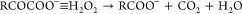
16

### Electrocatalysts for Organic-Nitrogen Oxidation in Real Waste Matrices

Nutrient recovery from complex matrices, such as wastewater sludge and livestock manure, can be achieved by the electrochemical oxidation of organic-N compounds. These matrices are composed of cells, particles, extracellular polymeric substances (EPS) such as phospholipids, and water. The resulting floc structure binds water in multiple ways and results in the presence of interstitial water (trapped within the floc structure), surface water (bound to the flocs), bound water (intercellular chemically bonded), as well as free water between flocs.^155,174^ The application of a potential within this complex matrix results in the dissolution of the sludge floc structure. This releases both the interstitial and bound water and disintegrates the membranes of the cells that are present within the sludge.^155^ Electrochemical treatment of sludge thus enhances dewaterability, removes pathogens and odors, and improves biodegradability during anaerobic digestion. As a result, biogas and VFA can be recovered more readily. Commonly used electrode materials to treat real sludge matrices include Ti-based catalysts, and well-understood materials like Ni and Fe are being explored for these applications.^59,64,156^

**Figure 6:**
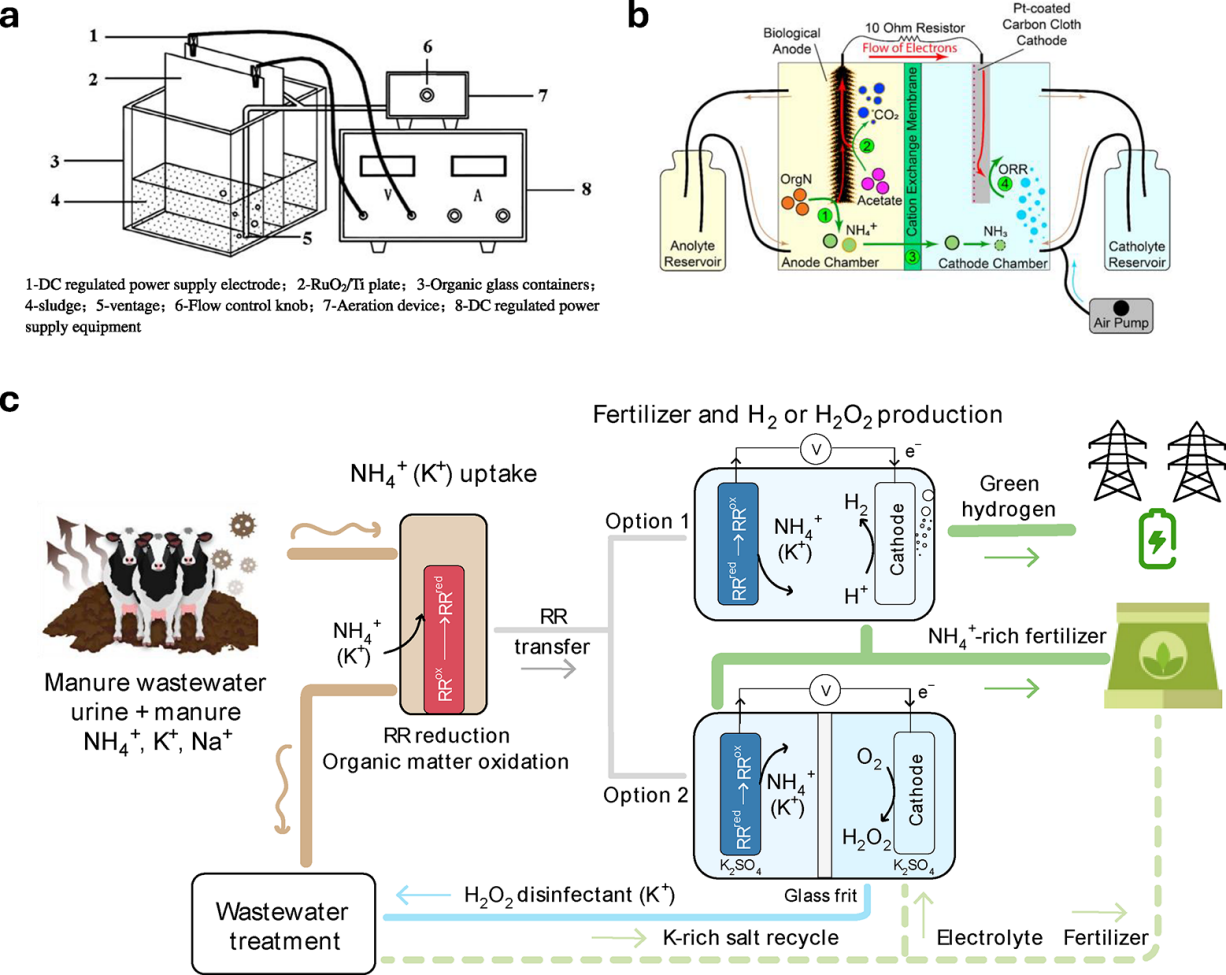
(a) Parallel plate electrochemical reactor for sludge pretreatment. Reprinted with permission from Yu et al.^176^ Copyright 2014 Elsevier. (b) Microbial fuel cell setup. Reprinted with permission from Burns et al.^182^ Copyright 2023 Elsevier. (c) NH_3_ recovery using NH_4_^+^ ion-selective redox reservoirs (RR) paired with electrochemical synthesis of NH_4_^+^-rich fertilizer. Reprinted with permission from Wang et al.^184^ Copyright 2023 Nature.

Ti-based catalysts successfully improve sludge dewaterability,^161,175^ volume reduction,^161^ and anaerobic digestion,^152,176^ but, overall, catalyst optimization for NH_3_ production has not been widely studied.^161,175,176^ Ti/RuO_2_ demonstrated good affinity toward sludge dewatering for large applied potentials, with 50 V for 5 min being the optimal conditions for dewatering while avoiding excessive proteins and polysaccharides being released from the EPS.^175^ The continued decrease in the reported sludge viscosity can be attributed to the released interstitial water, which is the desired outcome of the dewatering process, as the no-longer-bound water can be removed. In addition, the decrease in sludge viscosity was also in part due to the EPS degradation, but possible formed products were not measured. Electrocatalytic pretreatment using Ti/RuO_2_ mesh electrodes enhanced biogas production in an anaerobic digestion treatment process in the presence and absence of active Cl^–^,^176,177^ as well as increased the removal of volatile solids and volatile suspended solids in alkaline conditions (Figure 6a).^178^ Ti/Sb-SnO_2_/β-PbO_2_ was developed as an anode to carefully track the contributions toward sludge degradation from in situ formed active chlorine species as well as OH* and SO_4_^–^* radicals.^161^ Increases in total nitrogen content were attributed to cell breakage releasing intracellular nitrogenous compounds, and the addition of scavenger species (*tert*-butanol and methanol) indicated that OH* was the main contributor to the cell walls breaking. Graphite fiber placed on a Ti rod was shown to stabilize saline wastewater.^152^ Cell disintegration through the formed active Cl^–^ resulted in increased release of proteins and polysaccharides, which were found to decompose into small concentrations of NH_4_^+^ for applied potentials >5 V.

While Ni has been widely studied for the electrochemical oxidation of simple organic molecules, such as glycine and methanol,^179^ only recently Ni has been applied to treat more complex mixtures of organic-N. Ni is a catalyst to note due to its wide availability and Nickel easily forming surface oxides Ni(OH)_2_/NiOOH that can participate in organic oxidation reactions. Participation of NiOOH in waste-activated sludge oxidation has been associated with increased formation of NH_3_, in addition to other volatile fatty acids such as isobutyric acid and acetic acid.^154^ In the presence of Cl^–^, electroporation of the Ni surface can take place, resulting in Ni being dissolved into solution and so contributing to electrocoagulation of the organic species present.^180^ In a similar fashion, sacrificial Fe electrodes have been used to treat real textile wastewater using the electrochemical Fenton approach (Figure 5g).^165^ The addition of H_2_O_2_ was found to be the most favorable treatment process, in terms of chemical oxygen demand and color removal, when compared to electrocoagulation and electro-Fenton approaches. Stainless-steel electrodes have also been implemented for sludge pretreatment using pulsed voltammetry, where the subsequent breaking of the sludge structure helped solubilize the sludge components and increase the resulting methane production.^181^

Recent work on combined electrochemical approaches has had success in recovering NH_3_/NH_4_^+^ from manure wastestreams. In the field of bioelectrochemistry, newly proposed microbial fuel cells combine the established electrochemical separations of NH_4_^+^ from contaminated wastestreams with electrogenic microorganisms that mineralize organic N into NH_4_^+^ (Figure 6b).^182,183^ Carbon fiber brushes are used as supports for the selected exoelectrogenic microbial colony which can successfully mineralize the organic-N species to NH_4_^+^ as well as release electrons.^182^ Efficient conversion to NH_4_^+^ depends on the microbial strain present, and throughout the treatment process, the strains self-select to adapt to the changing microenvironment. Reaching low organic N concentrations results in the preferential degradation of microbial necromass (or the accumulated dead microbes) rather than the organic N, indicating a sensitivity to anolyte conditions. In the field of adsorbents, recent work combining electrochemical approaches with an ion-selective redox material has demonstrated NH_4_^+^ recovery from real manure wastewater with coproduction of H_2_O_2_ (Figure 6c).^184^ The developed KNiHCF material spontaneously oxidizes the organic matter and takes up the released NH_4_^+^ due to favorable intercalation properties. The saturated material can then be placed into a separate electrochemical reactor to behave as an anode, where NH_4_^+^ is released and can be recovered.

Urea can also be removed from real wastewater via an electrochemical process. Urea-formaldehyde-containing real wastewater from a medium-density fiberboard factory can be treated using Al electrodes. A total nitrogen removal of 76.7% was achieved in the electrochemical system. The Al anode dissociated into the solution reducing from 90% to 45%.^185^

## Product Analyses

Studies on electrochemical reactions involving inorganic nitrogen compounds, namely, NO_3_^–^, NO_2_^–^, and NH_3_ or NH_4_^+^, use different techniques to quantify the yielded products and byproducts. Moreover, the quantification of organic nitrogen species, such as amines, nitriles, and nitro compounds, generally involve the conversion of these organic nitrogen compounds into inorganic nitrogen via room-temperature chemical reactions, high temperature oxidation or photo-oxidation. Ion chromatography, spectrophotometric and colorimetric assays, fluorescence, and nuclear magnetic resonance (NMR) spectroscopy are the most widely used for detecting NH_3_ or NH_4_^+^ and oxyanions. Usually, more than one technique is used to validate the results. Ion chromatography provides higher sensitivity in comparison to spectrophotometric assays. However, ICs higher costs and the instrumentation required make spectrophotometric assays more widely used.^186^ Spectrophotometric assays are sensitive to pH levels and other ions and organic molecules present in the solution. These factors affect the accuracy of the product yields and efficiencies reported during catalyst testing. Although ion-specific testing for product quantification is recommended, common spectroscopy detection techniques of NH_3_ or NH_4_^+^, NO_2_^–^, and NO_3_^–^ are discussed in this section.

### Ammonia (NH_3_ or NH_4_^+^) Detection

**Figure 7:**
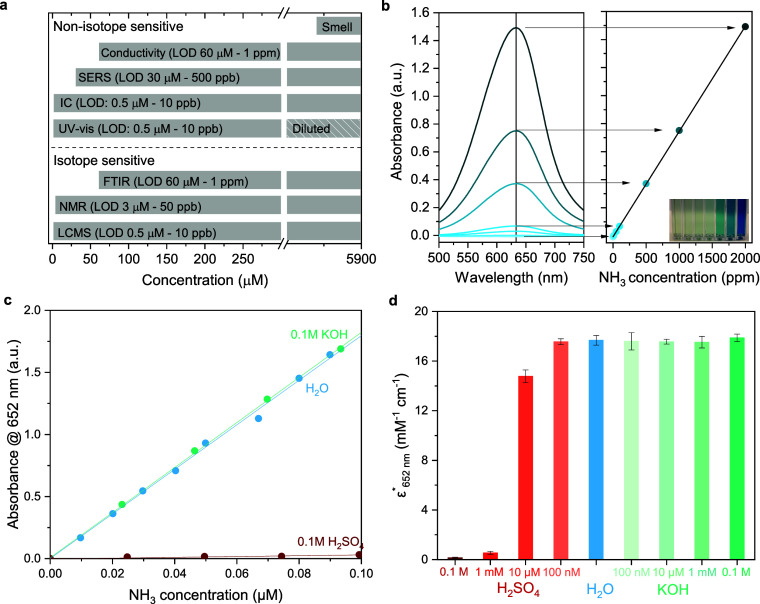
(a) Detection limits of well-known techniques for NH_3_ quantification including UV–visible light spectroscopy (UV–vis),^188^ conductivity meter measurements,^194^ nuclear magnetic resonance (NMR),^188^ Fourier transform infrared spectroscopy (FTIR),^195^ liquid chromatography mass spectroscopy (LCMS),^193^ surface enhanced Raman spectroscopy (SERS),^196^ and ion chromatography (IC).^192^ Olfactory detection becomes possible at high enough ammonia concentration, marked at 5882 μM (100 ppm) of NH_3_ in liquid.^197^ (b) UV–vis spectra of indophenol blue method, showing quantification via calibrated samples from 10 to 2000 ppb NH_3_ in H_2_O. The straight line is fitted based on peak absorbance of each sample, displaying linearity with sample concentration.^189^ Reprinted with permission from Iriawan et al.^190^ Copyright 2021 Springer Nature. (c)–(d) The effect of pH on the indophenol blue method. (c) Ammonia calibration curves via salicylate method (indophenol blue) for acidic (H_2_SO_4_) and alkaline (KOH) solutions. (d) Effective molar attenuation coefficient (in mM^–1^ cm^–1^) of the salicylate method for ammonia quantification at different pH levels, obtained from the slope of the fitted lines in (c) and the optical path of the used cuvette (1 cm). Modified with permission from Giner-Sanz et al.^191^ Copyright 2021 Elsevier.

UV–visible light spectroscopy (UV–vis) allows the fast and easy quantification of NH_3_ through colorimetric reactions. A summary of NH_3_ and NH_4_^+^ detection methods are shown in Figure 7. UV–vis techniques can lead to a precise ammonia quantification with a detection limit down to 10 ppb (∼0.5 μM) NH_3_^187−191^ and can be on par with ion chromatography (IC),^192^ proton NMR,^188^ and mass spectroscopy techniques,^193^ although the latter two have the added benefits of being isotopically sensitive, allowing for mechanistic studies. Other commonly used techniques are conductivity meter measurements,^194^ Fourier transform infrared spectroscopy (FTIR),^195^ liquid chromatography mass spectroscopy (LCMS),^193^ or surface enhanced Raman spectroscopy (SERS).^196,197^

**Figure 8:**
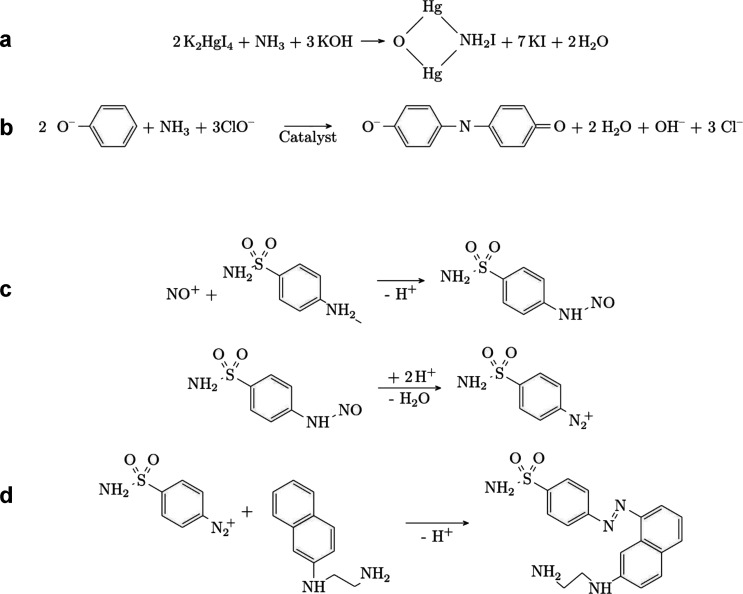
Diagram of the indophenol blue method reactions (a through b) and Griess method (c through e) for ammonia and nitrite detection.

In the case of NH_3_ detection, two colorimetric methods are used: (i) Nessler’s reagent and (ii) the indophenol blue method. Nessler’s reagent is a highly alkaline solution based on K_2_HgI_4_. The alkaline media are usually achieved using KOH (Figure 8a). This reacts with NH_3_ to develop a reddish brown color with absorbance signal detected at ∼420 nm.^198^ Nessler’s reagent was shown to be suitable when tested at a wide range of pH levels (from 4 to 12). On the other hand, the indophenol blue method presented an underestimate of NH_3_ concentration in acidic media, but was more exact on neutral and alkaline media. In addition, 0.01 mmol L^–1^ of different ions showed overestimates of the NH_3_ concentration when Nessler’s reagent was used, with Ru^3+^, Ce^3+^, and Fe^2+^ resulting in the biggest distortion in the results. The interference effect of the ions was explained by the absorption of some ions such as Ru^3+^, In^3+^, and Fe^2+^ and chemical reactions between Nessler’s reagent and ions such as Ni^2+^, In^3+^, and Fe^2+^.^186^

The indophenol blue method is based on the Berthelot reaction (Figure 8a). Here, NH_3_ reacts with phenol and hypochlorite in an alkaline solution to generate a blue indophenol product. Sodium nitroprusside acts as catalyst to intensify the product’s color, and a buffer is used to stabilize the pH. This results in a highly conjugated group which strongly absorbs light between 630 and 720 nm (Figure 8b), whose intensity can be correlated with the amount of NH_3_. In these cases, NH_3_ is the limiting reagent, while other reagents are in large excess. An example of a calibration curve is shown in Figure 7b, where a known NH_3_ concentration from ammonium chloride (NH_4_Cl) standard solutions is linearly correlated to the blue color absorbance. The alkaline pH of the reactions converts NH_4_^+^ into NH_3_, owing to p*K*_a,NH3_ of 9.2.^199^ A more widely used variant of the indophenol blue method is the salicylate method, where salicylic acid or sodium salicylate is used as the phenolic compound. The salicylate method has the benefit of avoiding toxic reagents^200,201^ such as phenol or *ortho*-chlorophenol fumes generated in the indophenol method and mercury salts in the Nessler method. Despite the simplicity of these techniques, several factors may influence the color development and hence the accuracy of these measurements, most notably the reaction time, exposure to light, solution pH, and interference from ions (Fe^3+^, Co^2+^ to S^2–^, etc.) and other organic species (e.g., methanol, carbonates) in the media.^198^

By tracking the indophenol dye intensity as a function of time, the blue color intensity is found to increase rapidly within the first 20 min and remains stable within 2 h. Beyond that, the indophenol dye will decompose, while a red coloration develops. The indophenol dye stability can be extended to longer than 24 h by protecting the solution from carbon dioxide absorption and direct sunlight.^202^ However, quantification within 2 h of initiating the reaction is recommended to ensure reproducibility.

The efficiency of indophenol dye development is also affected by solution pH.^198^ Acidic solutions lead to poor color development as indicated by the small slope in the calibration curve (Figure 7c) as well as the effective molar attenuation coefficient (Figure 7d, obtained from the slope of the calibration curve and the optical path of the used cuvette, in units of mM^–1^ cm^–1^) which indicates the ammonia quantification ability.^191^ The near-zero attenuation coefficient for acidic solutions (>1 mM H_2_SO_4_) is attributed to the precipitation of the salicylic acid where the pH is lower than the p*K*_a_ of the salicylic acid–salicylate base pair (2.97).^191^ This greatly reduces the availability of salicylate ion to participate in the color development reaction according to Krom’s mechanism,^203^ in which each NH_3_ overall reacts with two salicylate ions via oxidative coupling to form the dye. It is recommended that solutions with pH < 6 are neutralized (e.g., by adding KOH) prior to applying the salicylate method, while ensuring that the dilution factor is taken into account from the addition of KOH.

Moreover, interference effects from ions can significantly impact the accuracy of the colorimetric methods. This is especially pertinent because transition metal ions as a result of catalyst corrosion can occur^204^ and because Fe concentrations can exist up to 7 ppm (mg L^–1^) in real wastewater.^205^ In Nessler’s reagent, 0.01 mmol^–1^ of different ions are shown to overestimate NH_3_ concentration, Ru^3+^, Ce^3+^, and Fe^2+^ being the ones with that caused the biggest distortion in the results.^198,206^ Nevertheless, some of these can be overcome by using the Seignette reagent (also known as Rochelle salt), which allows the analysis of samples with high salinity.^206^ Fe^3+^ interference is also well-studied for the salicylate indophenol blue but found to have the opposite effect, where the Fe ions suppress the dye peak in the UV–vis spectra.^207^ To overcome this issue, a convenient methodology to correct the effect of strong Fe^3+^ interference by using an interference model requiring only three experimental curves has been reported.^207^

Strong interference from organic contaminants also needs to be taken into account, especially in the electrolysis of realistic wastewater. In Nessler’s reagent, organic solvents such as methanol, formaldehyde, formic acid, ethanol, acetone, dimethylformamide (DMF), dimethyl sulfoxide (DMSO), and triethanolamine have been shown to severely under or overestimate NH_3_ concentrations by up to a factor of 50×.^198^ In the indophenol blue method, compounds such as tetrahydrofuran, ethanol, and carbonates can lead to significant errors.

Real waste waters from upstream and agricultural sources can contain a meaningful amount of organic nitrogen, which constitutes up to 10% of total organic matter in water bodies.^208^ Specifically, amino acids whose amine groups can interact with the hypochlorite solution in the indophenol method lead to either a false positive or negative result. When the amino acid is present in small concentrations, it can emulate the role of NH_3_ in the blue color development,^209^ leading to a false positive result. The amine group in amino acids can also induce a nucleophilic attack toward the indophenol product, leading to a blue signal depression and possible false negative results.^210^ Several mitigation strategies can be effective, such as the removal of the organic compounds (e.g., via chromatographic techniques) or evaporation–redissolution process, where HCl is added to the analyte, followed by vacuum evaporation and redissolution of the crystals in water for colorimetric analysis.^191^ Flow-based methodologies can also be used to separate NH_3_ from the organics-containing matrices, where NH_3_ in gaseous form (e.g., via basification of the analyte) can cross a gas-permeable membrane and be captured on the other side for analysis.^211^ While these strategies can be applied, there are two ways in which the error of the quantification can be reduced: (i) generating calibration curves based on solutions that mimic the real analyte composition as closely as possible and (ii) at least two independent detection methods must show quantitative agreement.

### Nitrite (NO_2_^–^) Detection

The Griess method (Figures 8c through e) is widely used for colorimetric NO_2_^–^ detection in water treatment, photocatalysis, and electrocatalysis.^212^ Usually, a coloring solution is made with the reagents needed for the reaction to occur: sulfanilamide, *N*-(1-naphthyl)ethylenediamine, as well as phosphoric acid. This coloring solution is then added to the sample for measurement. Acidic conditions are important for the Griess method, especially for the NO^+^ formation and protonation of *N*-nitrosamine to the diazonium salt (Figure 8d). Then, it is expected for the pH of the sample to affect this colorimetric method.

To explore this effect, UV–vis spectra were measured using three electrolytes with different pH values used in the literature: 0.1 M KOH (pH 13), 0.1 M Na_2_SO_4_ (pH 7), and 0.05 M H_2_SO_4_ (pH 1). Tests in alkaline media demonstrated that the dye is not properly formed under these conditions, especially at NO_2_^–^ concentrations lower than 0.2 ppm. The resulting coloration is a pale red color for concentrations higher than 0.2 ppm (Figure S2). The difference in coloration results in a maximum absorbance at a wavelength around 555 nm (with 0.4 ppm), compared to a wavelength of 540 nm usually reported in the literature. In the case of neutral and acidic media, the coloration showed the distinct fuchsia dye color (Figure S2).

**Figure 9:**
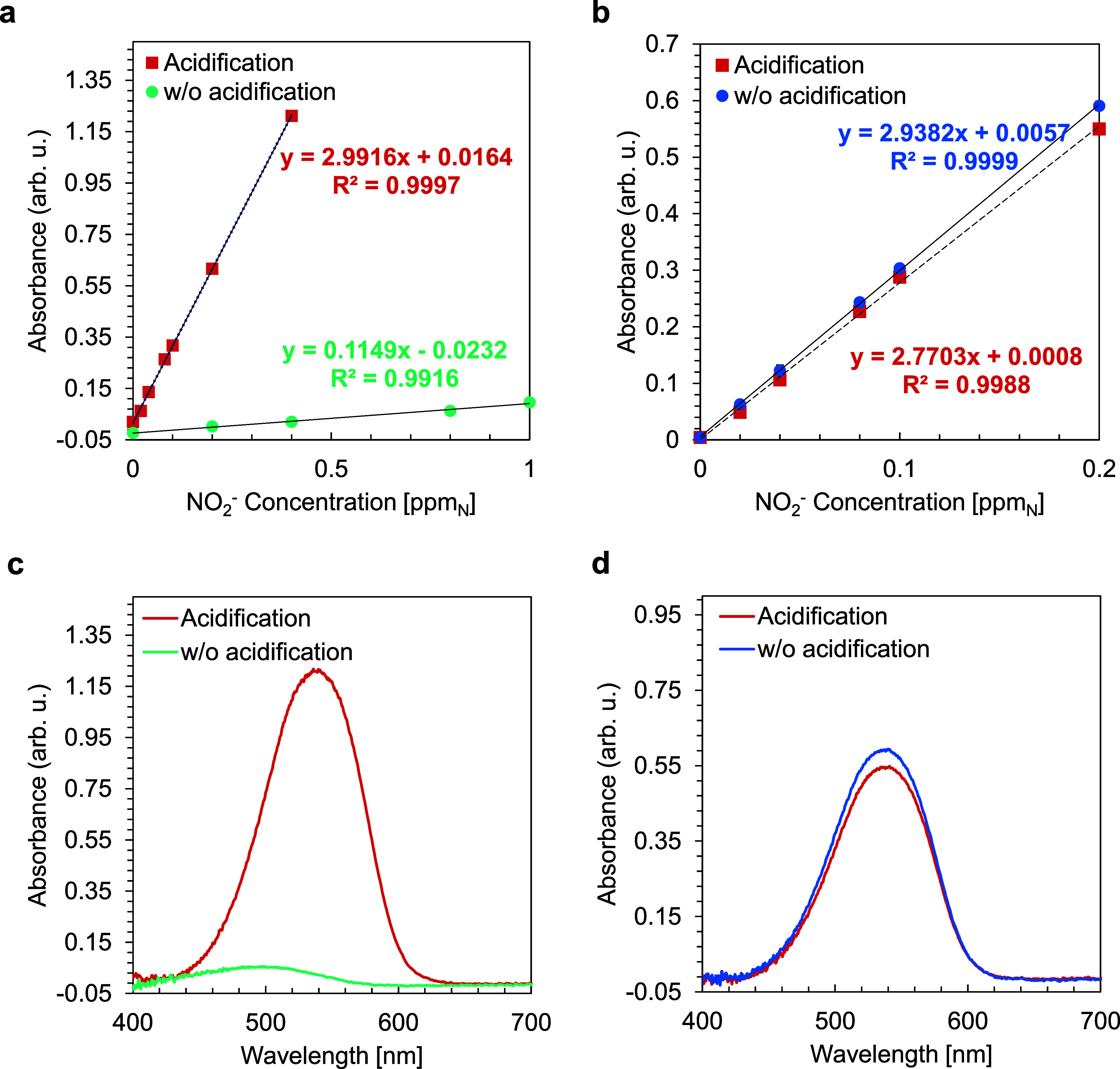
Effect of pH of the solution on nitrite colorimetric analysis under (a) 0.1 M KOH alkaline and (b) 0.1 M Na_2_SO_4_ neutral media with and without acidification. Absorbance spectra at (c) 0.4 ppm in 0.1 M KOH alkaline media with and without acidification and (d) 0.2 ppm in 0.1 M Na_2_SO_4_ neutral media with and without acidification. Acidification is done using 1 M HCl.

To get the distinct fuchsia color, some methods acidify the sample before adding the coloring solution.^213^ This procedure was followed by adding 1 M HCl to the sample (5 mL) before adding the coloring solution. In the case of alkaline media, the acidification allowed the dye to form, resulting in the fuchsia coloration (Figure S2), which allowed the maximum absorbance to be found at around 540 nm. The absorbance detected was significantly higher than that detected without prior acidification (Figure 9). This led to a decrease in error during the measurement, which resulted in a higher R^2^ in the linearization of the calibration curve. In the case of neutral media, the acidification showed a decrease in absorbance, but the maximum was maintained at 540 nm. In summary, the Griess method requires acidic conditions to form the dye for colorimetric measurement properly. As these results suggest, the acidification of alkaline media helped in the dye formation.

Organic matter, particularly organic nitrogen in the form of proteins, can potentially cause reliability issues in using the Griess method to detect NO_2_^–^ in complex matrices. Studies on the reliability of the Griess method in biological matrices show that proteins, sugars, and vitamins in serum and plasma samples can act as positive or negative interferents.^214^ The presence of coagulants can also lead to false signals in the 540 nm region due to elastic scattering. Mitigation strategies such as chromatographic separation, ultrafiltration, or the use of anticoagulant chemicals to suppress coagulant effects are important for the accurate use of the Griess method. The efficacy of these strategies should be evaluated via the generation of a reliable calibration curve in a mimicking analyte composition as well as quantitative validation via an independent technique such as ion chromatography.

### Nitrate (NO_3_^–^) Detection

NO_3_^–^ can generally be detected directly or indirectly, i.e., via reduction of NO_3_^–^ into NO_2_^–^ using a suitable catalyst such as Zn powder^215^ or vanadium(III) chloride,^216,217^ followed by the Griess assay. However, the latter method should be used with caution as it suffers from a comparatively high limit of detection of 500 ppb (∼10 μM), interferences with Fe^3+^, Cu^2+^, S^2–^, or I^–^, and the need for careful control of reaction conditions such as temperature.^216^ In this section, we focus on direct NO_3_^–^ detection.

The most common used wavelength for the NO_3_^–^ absorbance using UV–vis is around 220 nm. However, several organic compounds, such as carbonates, phosphates, and organic carbon,^218,219^ as well as solution turbidity^220^ can also absorb this particular wavelength. Moreover, OH^–^ group in alkaline media can also lead to a strong absorption in NO_3_^–^ relevant regions (Figure S3). Thus, another measurement at 275 nm is taken to subtract it from the measurement taken at 220 nm. Due to the influence of pH in the measurement, the pH of the sample and the reference/blank solutions in UV–vis measurement must be the same (Figure S4). Another way to deal with hydroxide group interference is an acidification process. Usually, 0.1 mL of 1 M HCl is added to 5 mL of the sample solution to acidify it.^114^ Other protocols for alkaline media have added 1 mL of 1 M HCl in a 4 mL sample.^213^ Here, the solution pH is adjusted to ∼1.5 (i.e., adding 37 μL of 70% HCl into 3 mL of 0.1 M KOH analytes).^221^

**Figure 10:**
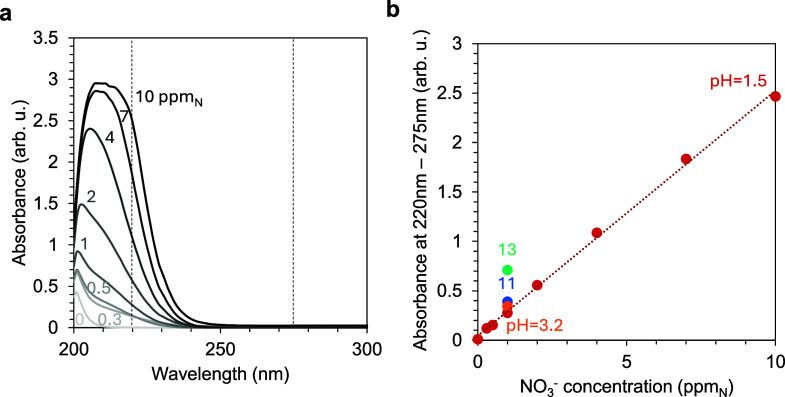
(a) UV–vis spectra and the calibration curves for NO_3_^–^ quantification, where the concentration of NO_3_^–^ standard can be correlated with the absorbance at 220–275 nm. The samples and blank have a pH of 1.5. (b) Calibration curve based on the (a) spectra. Samples of 1.0 ppm_N_ of NO_3_^–^ at pH 3.2, 11, and 13 are displayed to show the effect of pH changes in NO_3_^–^ quantification. The blank and sample solutions are pH-adjusted via the addition of HCl into KOH solutions. When the sample is more alkaline than the blank by ΔpH > 1, a significant overestimation in the UV–vis absorbance can be found. Methods and spectra at different pH can be found in Supporting Information.

As the OH^–^ group gives a strong interference, pH changes also affect the accuracy of NO_3_^–^ quantification. This is especially important as the cathodic compartment in electrochemical reduction experiments can become gradually more alkaline over time due to proton consumption (e.g., during H_2_ evolution). Figure 10 shows the sensitivity of the measurements to the change in pH, where the pH of the 1 ppm_N_ NO_3_^–^ solution has been gradually altered. When the pH values of the blank and sample differ by ΔpH > 1, the relative error in the absorbance value can be as large as 40%, suggesting that UV–vis technique must be used with caution especially when the knowledge is minimal concerning the electrolyte pH and its change over the period of electrolysis. In this case, IC is recommended due to its superior specificity toward NO_3_^–^.

Several organic compounds that possess bonds with vibrational frequencies similar to those of NO_3_^–^, such as NO_2_^–^, carbonates, and amino acids, can also interfere with the UV–vis measurement. Mitigating these interfering compounds typically involves the elimination of the interference compounds: NO_2_^–^ is usually reduced to N_2_ by using sulfamic acid. Moreover, transition metal ions can also interfere, such as Fe^3+^, which absorbs in the 200–280 nm range.^222^ One solution is by the removal of iron ions via raising the pH to precipitate Fe(OH)_3_ according to the Fe Pourbaix diagram^223^ followed by filtration, but the pH must be controlled upon such treatment given the significant influence of solution pH as discussed previously. When dealing with interfering compounds in the analytes, the efficacy of the mitigation strategies can be validated by using a second detection technique.

## Conclusion and Outlook

**Figure 11:**
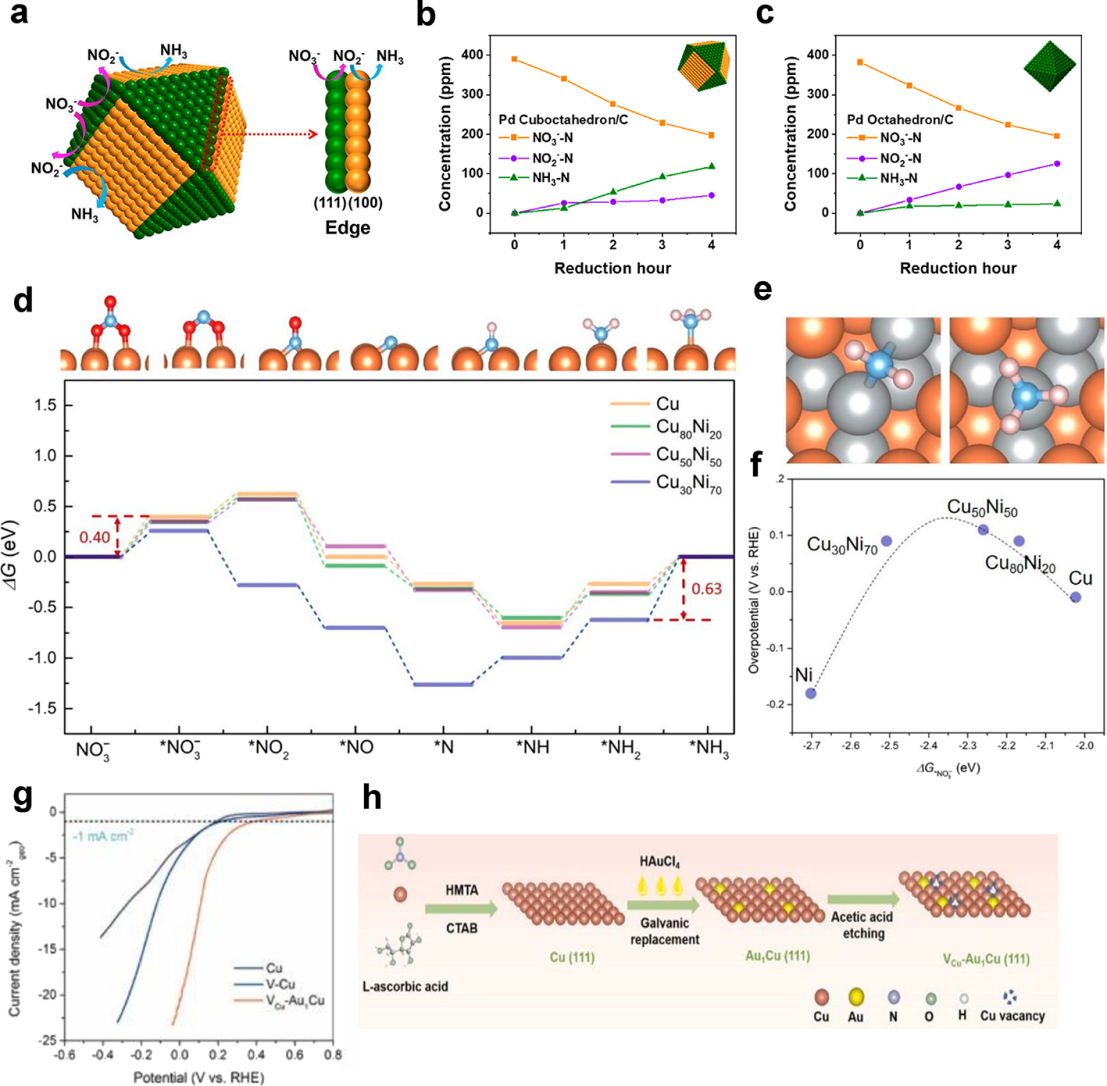
Different electrocatalyst design strategies. (a) through (c) show the importance of crystal facets. Reprinted in part with permission from Lim et al.^224^ Copyright 2021 ACS. (d) through (f) demonstrate the influence of alloying through density functional theory calculations. Reprinted with permission from Wang et al.^100^ Copyright 2020 ACS. (g) and (f) reveal the impact of defects on nitrate reduction. Reprinted in part with permission from Zhang et al.^225^ Copyright 2022 Elsevier. (a) Schematic showing that Pd(111) is selective for nitrate reduction toward nitrite and Pd(100) is selective toward ammonia production. Concentration change of NO_3_^–^-N, NO_2_^–^-N, and NH_3_-N per reaction time during chronoamperometry tests using an H-cell at −0.2 V vs RHE for (b) Pd cuboctahedron/C and (c) Pd octahedron/C. (d) Density functional theory calculations of reaction free energies for different intermediates on different CuNi surfaces. (e) Simulation of hydrogenation reaction of *NH_2_ (*NH_2_ + H_2_O + e^–^ → *NH_3_ + OH^–^) on a Cu_30_Ni_70_ surface. Red corresponds to oxygen atoms, pink to hydrogen atoms, blue to nitrogen atoms, gray to nickel atoms, and orange to copper atoms. (f) The volcano-type relationship between experimental overpotentials of NO_3_RR at 5 mA cm^–2^ in 10 mM KNO_3_ and adsorption energies of *NO_3_^–^ on different CuNi alloys. (g) Linear sweep voltammetry of Cu nanosheets, V_Cu_ nanosheets, and V_Cu_-Au_1_Cu single atom alloys tested in KNO_3_/KOH electrolyte. (h) Schematic diagram of V_Cu_-Au_1_Cu single atom alloy synthetic procedure.

Due to the environmental impact of carbon-based fuels and residuals generated during water treatment, electrochemical conversion of water contaminants into valuable chemicals such as NH_3_ or N_2_ is a promising alternative with several challenges to be addressed. The complexity of the NO_3_RR, NO_2_RR, and NO_2_OR requires catalyst design to selectively form NH_3_ or N_2_. Some strategies include alloying metals, using certain crystal facets,^224^ introducing defects, and finding appropriate supports for the catalyst (Figure 11). Alloying metals^101^ allows for the tuning of the d-orbital, which is relevant for the adsorption of reactants and intermediates.^77^ Atoms or molecules with a filled one-electron level below the d-bands show repulsive coupling of the d-states for noble metals if the antibonding peak is below the Fermi level.^226^ Therefore, it is relevant to tune the d-band in alloys to increase the d-band energy and decrease the filling of the antibonding states. In addition, alloys may change the morphology of the surface.^105,119^ Certain crystal facets of the same metal can show selectivity toward a particular product by stabilizing key intermediates. Defects like vacancies change the crystal lattice, create active sites, and help in the adsorption of reactants and intermediates or suppress competing reactions, as well as impinge the charge transfer.^88^ Support helps in the transport of reactants, avoids aggregation, and increases the conductivity. Other factors, such as the pH of the solution and the kinetics of the reaction, affect the performance of the catalyst. Overall, the conversion of NO_3_^–^ to NH_3_ and N_2_ has already been achieved with highly selective and stable electrocatalysts in lab-based prepared electrolytes supplemented with NO_3_^–^. In this context, metallic sites have been mainly identified as active sites and are stable under reductive conditions. Notably, the conversion to N_2_ has been sustained over long periods, with catalysts demonstrating exceptional stability.

When looking into real wastewater applications, these additional levers are important for optimizing reactor design for economic viability, as extensive catalyst engineering can be cost-prohibitive. Studies using more realistic wastewater electrolytes have also yielded the first successful tests. Co(DIM) catalysts for NO_3_^–^ reduction present in wastewater using reactive separations architecture reactors show that the presence of Mg^2+^ causes a decrease in NO_3_^–^ conversion by 62%. This may indicate a deactivation of the catalyst due to the Mg^2+^ present in the wastewater.^227^ The sources of instability of the catalysts remain unclear in most studies, as there has been a limited focus on catalyst and electrolyte characterization so far. Therefore, it is imperative to delve deeper into these areas in the future to strategically optimize the catalytic properties and cell setups. Finally, electrochemical sludge oxidation as treatment has great potential for value-added products such as NH_3_. This emerging field can be nourished by the findings in electrocatalytic reduction reactions.

## Supporting Information Available

Additional spectra and tables with data from figures presented in the text (PDF)
