# Supplementary material for: Electrocatalysts for Inorganic and Organic Waste Nitrogen Conversion
Source: ACS Catal. 2024 Jun 14;14(13):9752–75. doi: 10.1021/acscatal.4c01398 (PMC11232026; doi:10.1021/acscatal.4c01398)
Supplement: Supplementary file 1 — cs4c01398_si_001.pdf [file cs4c01398_si_001.pdf]

Electrocatalysts for Inorganic and Organic Waste Nitrogen Conversion

Supporting Information

Danae A. Chipoco Haro,[†] Luisa Barrera,[‡] Haldrian Iriawan,[¶] Antonia Herzog, [¶,§] Nianhan Tian, [‖] Andrew J. Medford, [‖] Yang Shao-Horn, [¶,§,∇] Faisal M. Alamgir[†], and Marta C. Hatzell[*,‡]

[†]School of Materials Science and Engineering, Georgia Institute of Technology, North Avenue 771 Ferst Dr., Atlanta, Georgia 30332, United States

[‡]George W. Woodruff School of Mechanical Engineering, Georgia Institute of Technology, 770 Ferst Ave, Atlanta, Georgia 30309, United States

[¶] Department of Materials Science & Engineering, Massachusetts Institute of Technology, 77 Massachusetts Avenue, Cambridge, Massachusetts, 02139, United States

[§] Research Laboratory of Electronics, Massachusetts Institute of Technology, 77 Massachusetts Avenue, Cambridge, Massachusetts 02139, United States

[‖] School of Chemical & Biomolecular Engineering, Georgia Institute of Technology, Atlanta, Georgia 30332, United States

[∇] Department of Mechanical Engineering, Massachusetts Institute of Technology, 77 Massachusetts Avenue, Cambridge, Massachusetts 02139, United States

E-mail: marta.hatzell@me.gatech.edu

Phone: +1 404-385-45036

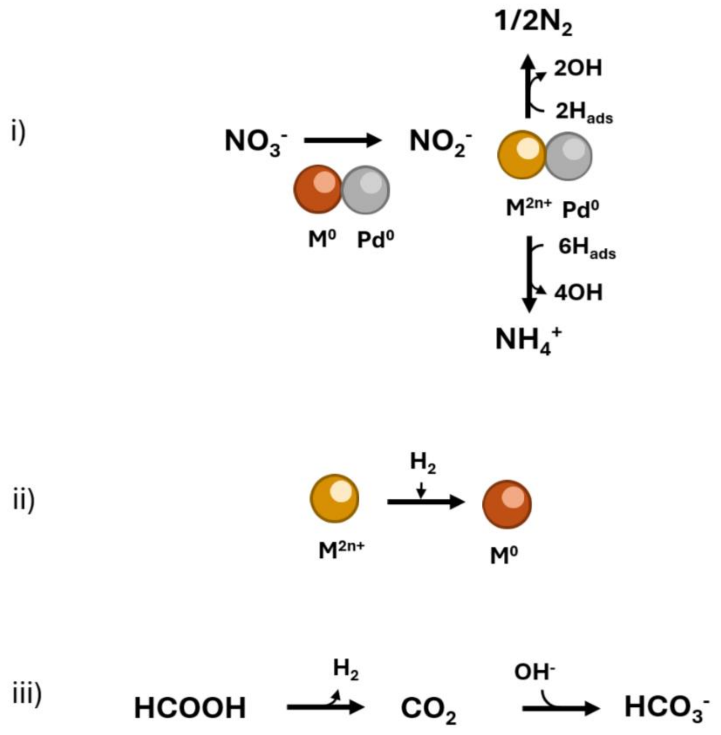

Figure S1. Catalytic reduction of NO_3^- (i) reduction of NO_3^- to NO_2^- and further reduction to NH_4^+ or N_2, (ii) catalyst regeneration, and (iii) pH neutralization using buffering agents because of pH change in the reaction.

NO_2^- UV-vis quantification

The color reagent was prepared by mixing 0.20 g of N-(1-naphthyl) ethylenediamine dihydrochloride (≥98%), 4.0 g of sulfonamide (≥99%) and 10ml of phosphoric acid (85 wt.% in H2O) with 50 ml of deionized water. In a test with acidification, 1ml HCl (1 M) was firstly added into the 5 ml of diluted post-electrolysis electrolytes, and then 0.1 ml of color reagent was added and shaken to obtain a uniform solution.

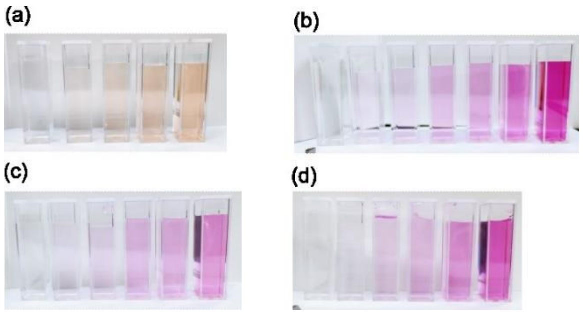

Figure S2. Dye formation using the Griess method under alkali conditions (a) without and (b) with acidification, and neutral condition (c) without and (d) with acidification.

NO_3^- UV-vis quantification

Calibration samples were made by dissolving the appropriate mass of KNO_3 (Sigma, ≥99.0%) into a 0.1M KOH solution made from potassium hydroxide pellets (Sigma, 99.99% trace metal basis) and Deionized water (Millipore, 18.2 M MΩ·cm at 25℃; TOC: 4 ppb) to make a 100 ppm_N NO_3^--N solution, which is then diluted down to the desired concentrations. For sample measurement, 3 mL of solution is used with a quartz cuvette. The as-is UV-vis spectra for the alkaline samples show large readings in the region of interest preventing quantification (Figure S3), so all 0.1M KOH samples are neutralized with strong acids, whereby 37 uL of 37% HCl (ACS reagent, Sigma) is added to 3 mL 0.1M KOH sample resulting in a solution pH 1.5. A GENESYS® 180 UV-Visible spectrophotometer was used to measure the UV-Vis spectra.

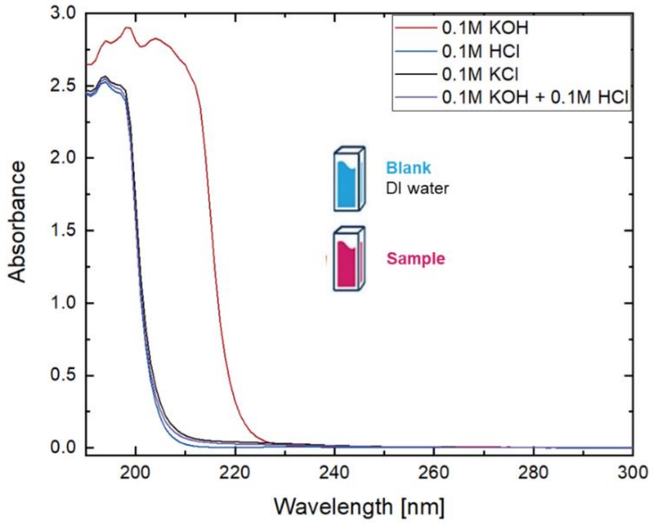

Figure S3. UV-vis spectra of 0.1M KOH (red), 0.1M HCl (blue), 0.1M KCl (black) and neutralization 0.1M KOH + 0.1M HCl (purple).

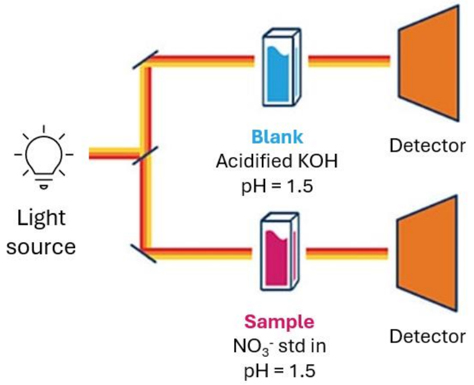

Figure S4. Schematic of the UV-vis setup for NO_3^- quantification, where both the blank and sample solutions are pH-adjusted to 1.5 via the addition of HCl into KOH solutions.

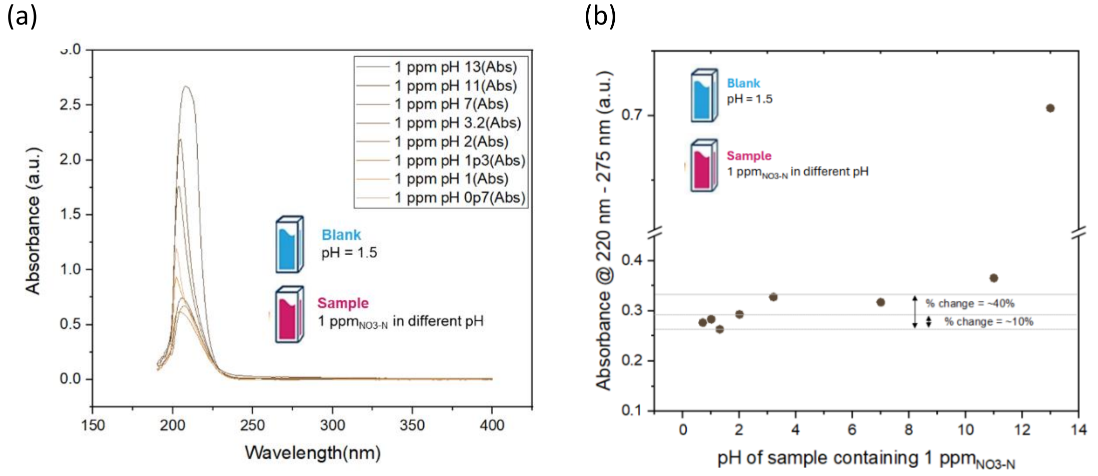

Figure S5. Effect of pH on NO_3^- quantification, where the blank solution is at pH =1.5 and the sample solution is 1 ppm_{-N} NO_3^- in different pHs from KOH and HCl adjustments. (a) spectra of 1 ppm_{-N} NO_3^- in different pHs and (b) quantification of 1 ppm_{-N} NO_3^- by subtracting the absorbance at 275 nm from the absorbance at 220 nm. The extracted NO_3^- quantification is plotted in Figure 7 of the main text.

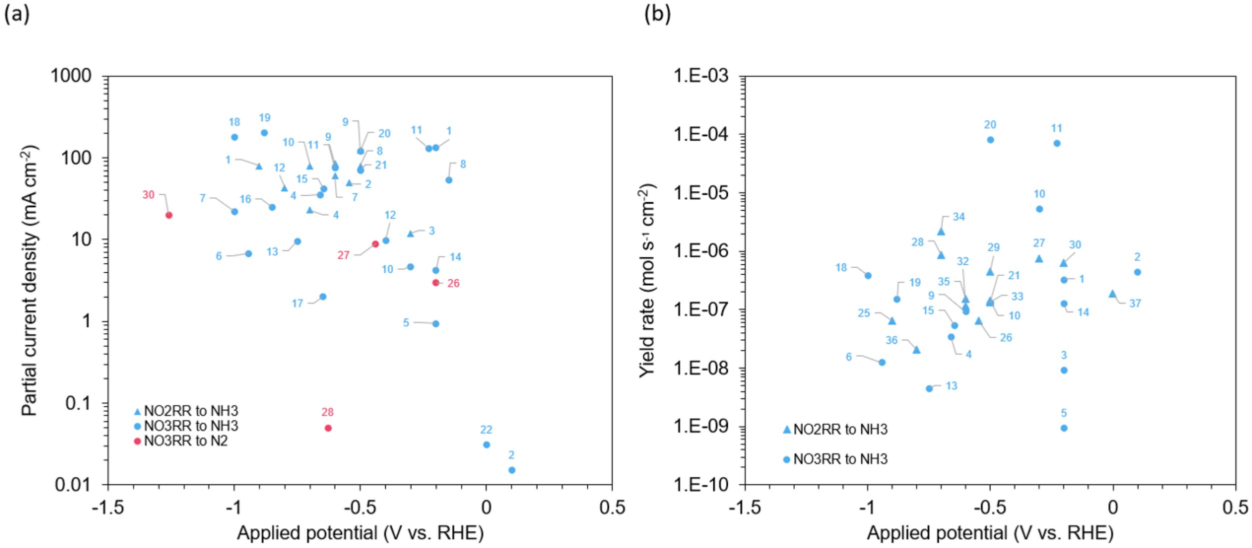

Figure S6. Literature overview of (a) partial current density and (b) yield rate of NO3RR and NO2RR. Specific details can be found on the Tables S1 through S4.

Table S1. Electrocatalysts for NO_3^- reduction reaction.

Electrocatalysts	Product	Potential (V vs RHE)	Partial current density (mA cm^{-2})	Yield rate (mol s^{-1} cm^{-2})	Faradaic efficiency (%)	Reference
Ru-ST-12	NH_3	-0.2	133	3.25E-07	100	[1]
$Pt_{78}Ru_{22}$/C	NH_3	0.1	0.015	4.444E-07	93	[2]
V_{Cu}-Au_1Cu	NH_3	-0.2	N/A	9.052E-09	98.7	[3]
Fe SAC (FeN_4)	NH_3	-0.6	35.3	3.422E-08	75	[4]
amorphous Ru/CNT	NH_3	-0.2	0.94*	9.466E-10	80.6	[5]
TiO_{2-x}	NH_3	-0.9	6.8*	1.25E-08	85	[6]
Ti foil	NH_3	-1	22	N/A	82	[7]
$Ni_{50}Cu_{50}$/PTFE	NH_3	-0.15	53	N/A	99	[8]
hallow Ti	NH_3	-0.6	75	9.167E-08	45	[9]
Co-P/Ti plate	NH_3	-0.3	4.7*	5.301E-06	93.6	[10]
Cu@Ni foam	NH_3	-0.2	131	7E-05	96.6	[11]
Pt-Cu foam	NH_3	-0.4	9.8	N/A	22	[12]
NbO_x	NH_3	-0.75	9*	4.485E-09	94.5	[13]
Pd octahedron/C	NH_3	-0.2	4.25	1.252E-07	35.1	[14]
Co/CoO nanosheet	NH_3	-0.646	40*	5.402E-09	93.8	[15]
MWCNT	NH_3	-0.85	25	N/A	73	[16]
F-C	NH_3	-0.65	2	N/A	20	[17]
Cu-N-C	NH_3	-1	180	3.833E-07	95.5	[18]
Fe SAC	NH_3	-0.88	200	1.501E-07	85	[19]
plasma CuO-5	NH_3	-0.5	120	8.056E-05	89	[20]
Fe3C/NC	NH_3	-0.5	69.5	1.322E-07	96.7	[21]
oxo-MoSx	NH_3	0	0.031	N/A	96	[22]
amorphous Ru/CNT	N_2	-0.2	3	N/A	17.48	[5]
NF-Cu/Pd	N_2	-0.4	8.9*	N/A	N/A	[23]
B-doped diamond	N_2	-0.6	0.05	N/A	45.2	[24]
CR B-doped diamond	N_2	-0.9	N/A	N/A	44.5	[24]

*Partial current density was calculated with the faradaic efficiency.

Table S2. Electrocatalysts for NO_2^- reduction reaction.

**Electrocatalysts**	**Product**	**Potential (V vs RHE)**	**Partial current density (mA cm-^{2})**	**Yield rate (mol s^{-1} cm^{-2})**	**Faradaic efficiency (%)**	**Reference**
CoB@TiO_2	NH_3	-0.9	80	6.475E-08	95.2 (at -0.7V)	[25]
Ni-NSA-V_{Ni}	NH_3	-0.5	49*	6.555E-08	88.9	[26]
Ni_2P/Ni foam	NH_3	-0.3	12*	7.478E-7	90.2	[27]
Ag@NiO/CC	NH_3	-0.4	23*	8.733E-07 (at -0.7V)	97.7	[28]
Cu_3P nanoarray/Cu foam	NH_3	-0.5	N/A	4.518E-07	91.2	[29]
CoP nanoarray	NH_3	-0.2	N/A	6.28E-07	90	[30]
[Co(DIM)Br_2]+	NH_3	-0.4	N/A	N/A	88	[31]
V-TiO_2/Ti plate	NH_3	-0.6	60	1.158E-07	93.2	[32]
NiS_2@TiO_2/Ti mesh	NH_3	-0.5	80	1.348E-07	92.1	[33]
Ag NPs@TiO_2 nanoribbon	NH_3	-0.5	122.1 (at -0.7 V)	1.428E-07	96.4	[10]
TiO_{2-x} NBA	NH_3	-0.7	80	2.193E-06	92.7	[34]
P-doped TiO_2	NH_3	-0.6	85*	1.557E-07	90	[35]
Ni NPS@C	NH_3	-0.9	43.13	2.055E-08 (at -0.8 V)	65.4	[36]
Ni_2P	NH_3	0	N/A	1.881E-07	92.6	[37]

*Partial current density was calculated with the faradaic efficiency.

Table S3. Electrocatalysts for NO_2^- oxidation.

Electrocatalysts	pH	LoD (μM)	Linear range (μM)	Sensitivity (μA mM^{-1} cm^{-2})	Reference
N,O co-doped graphene	4	0.8	N//A		[38]
Au/f-GE/GCE	7	0.01	0.125–20375.98		[39]
Au NRs–Nafion-modified GCE	7	0.64	3–6000	130.9	[40]
$LaAlO_3$@GO	7	0.0041	0.01-1540.5	1.132	[41]
Biofilm/carbon felt	7.5	2.39	25750	48.91	[42]
CuO/MWCNTs	7	0.039	0.1-1200	501	[42]
rGO/ZnO/Nafion/GCE	7.1	1.18	200-4000	315.6	[43]
TiO_2–Ti3C2TX/CTAB/chitosan	7	0.85	3-250	53	[44]
CSe_2 NF/CC	7	0.04	0.5-1800	2048.6	[45]
MoO_3/Co_3O_4	7	0.075	0.3125 - 4514	1704.1	[46]
MOF derived rod-like Co@C	N/A	0.17	0.5-4000<br>4000-8000	1.03	[47]
CoRuS NPs/B,N co-doped graphene	N/A	0.0159	0.001–1290	1.5309	[48]
AuNPs C Screen Printed	N/A	0.0025	0.01-4	0.85	[49]

Table S4. DFT studies on mechanisms for NO_3^- and NO_2^- reduction.

**Catalyst**	**Intermediate Adsorbed States Studied**	**Pseudopotentials**	**Exchange-Correlation Functional**	**Cutoff Energy (eV)**	**k-points**	**Reference**
Fe(310)<br>Co(211)<br>Ni(211)<br>Ag(211)<br>Au(211)<br>Cu(211)<br>Pt(211)<br>Rh(211)	*O, *OH, *H_2O, *NO_3, *NO_2, *N, *H, *NO, *N_2O, *N_2, *NH, *NH_2, *NH_3	Projector Augmented Wave (PAW)	Perdew–Burke–Ernzerhof (PBE)	400	2*4*1	[50]
Pt(111)<br>Pt(211)<br>Cu(111)<br>Cu(211)<br>Au(111)<br>Au(211)<br>Ag(111)<br>Ag(211)	*NO, *NOH, *HNOH, *NH, *cis-(NO-NO), *NO-NOH, *N_2O, *NH_2, *NH_3, *NH_4	Projector Augmented Wave (PAW)	Perdew–Burke–Ernzerhof (PBE)	400	4*4*1	[51]
Ru	*NO_3, *NO_2, *HNO_2, *NO, *HNO, *H_2NO, *NH_2, *NH_3	Projector Augmented Wave (PAW)	Perdew–Burke–Ernzerhof (PBE)	450	3*3*1	[1]
Fe Single Atom catalyst (SAC) on graphene	*NO_3, *HNO_3, *NO_2, *HNO_2, *NO, *HNO, *N, *NH, *NH_2, *NH_3	ultrasoft pseudopotentials (US-PPs)	Perdew–Burke–Ernzerhof (PBE)	500	4*4*1	[37]
Ru_{18} metal cluster	*NH_3, *NO_2, *NO, *N_2O, *NH_2, *NH_3, *H_2O, *H, *N_2	SDD effective core pseudo-potential	PBE0(IEF-PCM)/Lanl2DZ(Ru)/6-31+G(d,p)	N/A	N/A	[52]
Ru-doped anatase TiO_2 (101)	*NO_2, *NOOH, *NO, *NOH, *N, *NH, *NH_2, *NH_3	Projector Augmented Wave (PAW)	Perdew–Burke–Ernzerhof (PBE)	400	3*3*1	[53]
Ag(111)<br>Ag (100)	*NO_2, *NOOH, *NO, *NOH, *N, *NH, *NH_2, *NH_3	Projector Augmented Wave (PAW)	Perdew–Burke–Ernzerhof (PBE) with DFT+D3	450	3*3*1	[28]
Pd_xAg_{1-x}(111)	*N, *N_2		Perdew–Burke–Ernzerhof (PBE)	400	3*3*1	[54]
Cu<br>Cu/Cu_2O	*NO_3, *NO_2, *NO, *NOH, *NH_2OH, *NH_3	Projector Augmented Wave (PAW)	Perdew–Burke–Ernzerhof (PBE)	400	2*3*1	[55]
Cu_3P(300)	*NO_2, *NO, *NHO, *NHOH, *NH, *NH_2, *NH_3, *NO*OH	Projector Augmented Wave (PAW)	Perdew–Burke–Ernzerhof (PBE) with DFT+D3	450	5*5*1	[29]
Fe SAC (FeN_2O_2)	*NO_3, *NO_2OH, *NO_2, *NO_2H, *NO,	Projector Augmented Wave (PAW)	Perdew–Burke–Ernzerhof (PBE)	450	2*2*1	[19]

	*NOH, *N, *NH, *NH_2, *NH_3					
TiO_2(101) V–TiO_2(101)	*NO_2, *NO_2H, *NO, *ONH, *ONH_2, *NH_3, *O, *OH		Perdew–Burke–Ernzerhof (PBE)		3*3*1	[32]
TiO_2 (101)	*NO_2, *NO, *N, *NH, *NH_2, *NH_3	Projector Augmented Wave (PAW)	Perdew–Burke–Ernzerhof (PBE)	500	2*2*1	[35]
Ni_2P Ni_2P-H	*NO_2, *HNO_2, *NO, *H_2NO, *H_2NOH, *NH_3		Local Density Approximation (LDA)	340	0.04/ $Å^3$	[37]
Ni_2P(111) Ni_2P (201) Ni_2P (210)	*NO_2, *NH_3, *NH_2, *NH, *N, *NOH, *NO, *NO_2H.	Projector Augmented Wave (PAW)	Perdew–Burke–Ernzerhof (PBE) with DFT+D3	450	4*4*1	[27]
CoP(112)	*NO_2, *NO_2H, *ON, *OHN, *N, *NH, *NH_2, *NH_3	Projector Augmented Wave (PAW)	Perdew–Burke–Ernzerhof (PBE) with DFT+D3	450	4*3*1	[30]
Ni(111) Ni(200)	*NO_2, *NO_2H, *NO, *NOH, *N, *NH, *NH_2, *NH_3	Projector Augmented Wave (PAW)	Perdew–Burke–Ernzerhof (PBE) with DFT+D3	500	3*3*1	[56]
CoP(211)	*NO_3, *NO_2OH, *NO_2, *NOOH, *NO, *NOH, *N, *NH, *NH_2, *N_2O_3, *N_2O_2OH, *N_2O_2, *N_2OOH, *N_2O, *N_2OH, *N_2	Projector Augmented Wave (PAW)	Perdew–Burke–Ernzerhof (PBE)	450	7*7*7	[57]

References

(1) Li, J.; Zhan, G.; Yang, J.; Quan, F.; Mao, C.; Liu, Y.; Wang, B.; Lei, F.; Li, L.; Chan, A. W. M.; Xu, L.; Shi, Y.; Du, Y.; Hao, W.; Wong, P. K.; Wang, J.; Dou, S.-X.; Zhang, L.; Yu, J. C. Efficient Ammonia Electrosynthesis from Nitrate on Strained Ruthenium Nanoclusters. *J. Am. Chem. Soc.* **2020**, *142* (15), 7036–7046. https://doi.org/10.1021/jacs.0c00418.

(2) *Comparing electrocatalytic and thermocatalytic conversion of nitrate on platinum–ruthenium alloys - Catalysis Science & Technology (RSC Publishing) DOI:10.1039/D1CY01075A*. https://pubs.rsc.org/en/content/articlehtml/2021/cy/d1cy01075a (accessed 2023-09-01).

(3) Zhang, Y.; Chen, X.; Wang, W.; Yin, L.; Crittenden, J. C. Electrocatalytic Nitrate Reduction to Ammonia on Defective Au1Cu (111) Single-Atom Alloys. *Appl. Catal. B Environ.* **2022**, *310*, 121346. https://doi.org/10.1016/j.apcatb.2022.121346.

(4) Wu, Z.-Y.; Karamad, M.; Yong, X.; Huang, Q.; Cullen, D. A.; Zhu, P.; Xia, C.; Xiao, Q.; Shakouri, M.; Chen, F.-Y.; Kim, J. Y. (Timothy); Xia, Y.; Heck, K.; Hu, Y.; Wong, M. S.; Li, Q.; Gates, I.; Siahrostami, S.; Wang, H. Electrochemical Ammonia Synthesis via Nitrate Reduction on Fe Single Atom Catalyst. *Nat. Commun.* **2021**, *12* (1), 2870. https://doi.org/10.1038/s41467-021-23115-x.

(5) Jiang, M.; Tao, A.; Hu, Y.; Wang, L.; Zhang, K.; Song, X.; Yan, W.; Tie, Z.; Jin, Z. Crystalline Modulation Engineering of Ru Nanoclusters for Boosting Ammonia Electrosynthesis from Dinitrogen or Nitrate. *ACS Appl. Mater. Interfaces* **2022**, *14* (15), 17470–17478. https://doi.org/10.1021/acsami.2c02048.

(6) Jia, R.; Wang, Y.; Wang, C.; Ling, Y.; Yu, Y.; Zhang, B. Boosting Selective Nitrate Electroreduction to Ammonium by Constructing Oxygen Vacancies in TiO2. *ACS Catal.* **2020**, *10* (6), 3533–3540. https://doi.org/10.1021/acscatal.9b05260.

(7) McEnaney, J. M.; Blair, S. J.; Nielander, A. C.; Schwalbe, J. A.; Koshy, D. M.; Cargnello, M.; Jaramillo, T. F. Electrolyte Engineering for Efficient Electrochemical Nitrate Reduction to Ammonia on a Titanium Electrode. *ACS Sustain. Chem. Eng.* **2020**, *8* (7), 2672–2681. https://doi.org/10.1021/acssuschemeng.9b05983.

(8) Wang, Y.; Xu, A.; Wang, Z.; Huang, L.; Li, J.; Li, F.; Wicks, J.; Luo, M.; Nam, D.-H.; Tan, C.-S.; Ding, Y.; Wu, J.; Lum, Y.; Dinh, C.-T.; Sinton, D.; Zheng, G.; Sargent, E. H. Enhanced Nitrate-to-Ammonia Activity on Copper–Nickel Alloys via Tuning of Intermediate Adsorption. *J. Am. Chem. Soc.* **2020**, *142* (12), 5702–5708. https://doi.org/10.1021/jacs.9b13347.

(9) Krzywda, P. M.; Rodríguez, A. P.; Cino, L.; Benes, N. E.; Mei, B. T.; Mul, G. Electroreduction of NO3− on Tubular Porous Ti Electrodes. *Catal. Sci. Technol.* **2022**, *12* (10), 3281–3288. https://doi.org/10.1039/D2CY00289B.

(10) Fan, X.; He, X.; Ji, X.; Zhang, L.; Li, J.; Hu, L.; Li, X.; Sun, S.; Zheng, D.; Luo, Y.; Wang, Y.; Xie, L.; Liu, Q.; Ying, B.; Sun, X. High-Efficiency Electrosynthesis of Ammonia with Selective Reduction of Nitrite over Ag Nanoparticles-Decorated TiO2 Nanoribbon Array. *Inorg. Chem. Front.* **2023**. https://doi.org/10.1039/D2QI02409H.

(11) Li, J.; Gao, J.; Feng, T.; Zhang, H.; Liu, D.; Zhang, C.; Huang, S.; Wang, C.; Du, F.; Li, C.; Guo, C. Effect of Supporting Matrixes on Performance of Copper Catalysts in Electrochemical Nitrate Reduction to Ammonia. *J. Power Sources* **2021**, *511*, 230463. https://doi.org/10.1016/j.jpowsour.2021.230463.

(12) Cerrón-Calle, G. A.; Fajardo, A. S.; Sánchez-Sánchez, C. M.; Garcia-Segura, S. Highly Reactive Cu-Pt Bimetallic 3D-Electrocatalyst for Selective Nitrate Reduction to Ammonia. *Appl. Catal. B Environ.* **2022**, *302*, 120844. https://doi.org/10.1016/j.apcatb.2021.120844.

(13) Wan, X.; Guo, W.; Dong, X.; Wu, H.; Sun, X.; Chu, M.; Han, S.; Zhai, J.; Xia, W.; Jia, S.; He, M.; Han, B. Boosting Nitrate Electroreduction to Ammonia on NbO x via Constructing Oxygen Vacancies. *Green Chem.* **2022**, *24* (3), 1090–1095. https://doi.org/10.1039/D1GC04483D.

(14) Lim, J.; Liu, C.-Y.; Park, J.; Liu, Y.-H.; Senftle, T. P.; Lee, S. W.; Hatzell, M. C. Structure Sensitivity of Pd Facets for Enhanced Electrochemical Nitrate Reduction to Ammonia. *ACS Catal.* **2021**, *11* (12), 7568–7577. https://doi.org/10.1021/acscatal.1c01413.
(15) Yu, Y.; Wang, C.; Yu, Y.; Wang, Y.; Zhang, B. Promoting Selective Electroreduction of Nitrates to Ammonia over Electron-Deficient Co Modulated by Rectifying Schottky Contacts. *Sci. China Chem.* **2020**, *63* (10), 1469–1476. https://doi.org/10.1007/s11426-020-9795-x.
(16) Harmon, N. J.; Rooney, C. L.; Tao, Z.; Shang, B.; Raychaudhuri, N.; Choi, C.; Li, H.; Wang, H. Intrinsic Catalytic Activity of Carbon Nanotubes for Electrochemical Nitrate Reduction. *ACS Catal.* **2022**, *12* (15), 9135–9142. https://doi.org/10.1021/acscatal.2c01144.
(17) Li, Y.; Xiao, S.; Li, X.; Chang, C.; Xie, M.; Xu, J.; Yang, Z. A Robust Metal-Free Electrocatalyst for Nitrate Reduction Reaction to Synthesize Ammonia. *Mater. Today Phys.* **2021**, *19*, 100431. https://doi.org/10.1016/j.mtphys.2021.100431.
(18) Xu, M.; Xie, Q.; Duan, D.; Zhang, Y.; Zhou, Y.; Zhou, H.; Li, X.; Wang, Y.; Gao, P.; Ye, W. Atomically Dispersed Cu Sites on Dual-Mesoporous N-Doped Carbon for Efficient Ammonia Electrosynthesis from Nitrate. *ChemSusChem* **2022**, *15* (11), e202200231. https://doi.org/10.1002/cssc.202200231.
(19) Zhang, W.-D.; Dong, H.; Zhou, L.; Xu, H.; Wang, H.-R.; Yan, X.; Jiang, Y.; Zhang, J.; Gu, Z.-G. Fe Single-Atom Catalysts with Pre-Organized Coordination Structure for Efficient Electrochemical Nitrate Reduction to Ammonia. *Appl. Catal. B Environ.* **2022**, *317*, 121750. https://doi.org/10.1016/j.apcatb.2022.121750.
(20) Daiyan, R.; Tran-Phu, T.; Kumar, P.; Iputera, K.; Tong, Z.; Leverett, J.; Khan, M. H. A.; Esmailpour, A. A.; Jalili, A.; Lim, M.; Tricoli, A.; Liu, R.-S.; Lu, X.; Lovell, E.; Amal, R. Nitrate Reduction to Ammonium: From CuO Defect Engineering to Waste NOx-to-NH3 Economic Feasibility. *Energy Environ. Sci.* **2021**, *14* (6), 3588–3598. https://doi.org/10.1039/D1EE00594D.
(21) Wang, Y.; Zhang, L.; Niu, Y.; Fang, D.; Wang, J.; Su, Q.; Wang, C. Boosting NH3 Production from Nitrate Electroreduction via Electronic Structure Engineering of Fe3C Nanoflakes. *Green Chem.* **2021**, *23* (19), 7594–7608. https://doi.org/10.1039/D1GC01913A.
(22) Li, Y.; Go, Y. K.; Ooka, H.; He, D.; Jin, F.; Kim, S. H.; Nakamura, R. Enzyme Mimetic Active Intermediates for Nitrate Reduction in Neutral Aqueous Media. *Angew. Chem. Int. Ed.* **2020**, *59* (24), 9744–9750. https://doi.org/10.1002/anie.202002647.
(23) Shen, Z.; Liu, D.; Peng, G.; Ma, Y.; Li, J.; Shi, J.; Peng, J.; Ding, L. Electrocatalytic Reduction of Nitrate in Water Using Cu/Pd Modified Ni Foam Cathode: High Nitrate Removal Efficiency and N2-Selectivity. *Sep. Purif. Technol.* **2020**, *241*, 116743. https://doi.org/10.1016/j.seppur.2020.116743.
(24) Kuang, P.; Natsui, K.; Einaga, Y. Comparison of Performance between Boron-Doped Diamond and Copper Electrodes for Selective Nitrogen Gas Formation by the Electrochemical Reduction of Nitrate. *Chemosphere* **2018**, *210*, 524–530. https://doi.org/10.1016/j.chemosphere.2018.07.039.
(25) Hu, L.; Zhao, D.; Liu, C.; Liang, Y.; Zheng, D.; Sun, S.; Li, Q.; Liu, Q.; Luo, Y.; Liao, Y.; Xie, L.; Sun, X. Amorphous CoB Nanoarray as a High-Efficiency Electrocatalyst for Nitrite Reduction to Ammonia. *Inorg. Chem. Front.* **2022**, *9* (23), 6075–6079. https://doi.org/10.1039/D2QI01363K.
(26) Wang, C.; Zhou, W.; Sun, Z.; Wang, Y.; Zhang, B.; Yu, Y. Integrated Selective Nitrite Reduction to Ammonia with Tetrahydroisoquinoline Semi-Dehydrogenation over a Vacancy-Rich Ni Bifunctional Electrode. *J. Mater. Chem. A* **2021**, *9* (1), 239–243. https://doi.org/10.1039/D0TA09590G.
(27) Wen, G.; Liang, J.; Zhang, L.; Li, T.; Liu, Q.; An, X.; Shi, X.; Liu, Y.; Gao, S.; Asiri, A. M.; Luo, Y.; Kong, Q.; Sun, X. Ni2P Nanosheet Array for High-Efficiency Electrohydrogenation of Nitrite to Ammonia at Ambient Conditions. *J. Colloid Interface Sci.* **2022**, *606*, 1055–1063. https://doi.org/10.1016/j.jcis.2021.08.050.
(28) Liu, Q.; Wen, G.; Zhao, D.; Xie, L.; Sun, S.; Zhang, L.; Luo, Y.; Ali Alshehri, A.; Hamdy, M. S.; Kong, Q.; Sun, X. Nitrite Reduction over Ag Nanoarray Electrocatalyst for Ammonia Synthesis. *J. Colloid Interface Sci.* **2022**, *623*, 513–519. https://doi.org/10.1016/j.jcis.2022.04.173.

(29) Liang, J.; Deng, B.; Liu, Q.; Wen, G.; Liu, Q.; Li, T.; Luo, Y.; Alshehri, A. A.; Alzahrani, K. A.; Ma, D.; Sun, X. High-Efficiency Electrochemical Nitrite Reduction to Ammonium Using a Cu3P Nanowire Array under Ambient Conditions. *Green Chem.* **2021**, *23* (15), 5487–5493. https://doi.org/10.1039/D1GC01614H.
(30) Wen, G.; Liang, J.; Liu, Q.; Li, T.; An, X.; Zhang, F.; Alshehri, A. A.; Alzahrani, K. A.; Luo, Y.; Kong, Q.; Sun, X. Ambient Ammonia Production via Electrocatalytic Nitrite Reduction Catalyzed by a CoP Nanoarray. *Nano Res.* **2022**, *15* (2), 972–977. https://doi.org/10.1007/s12274-021-3583-9.
(31) Xu, S.; Kwon, H.-Y.; Ashley, D. C.; Chen, C.-H.; Jakubikova, E.; Smith, J. M. Intramolecular Hydrogen Bonding Facilitates Electrocatalytic Reduction of Nitrite in Aqueous Solutions. *Inorg. Chem.* **2019**, *58* (14), 9443–9451. https://doi.org/10.1021/acs.inorgchem.9b01274.
(32) Wang, H.; Zhang, F.; Jin, M.; Zhao, D.; Fan, X.; Li, Z.; Luo, Y.; Zheng, D.; Li, T.; Wang, Y.; Ying, B.; Sun, S.; Liu, Q.; Liu, X.; Sun, X. V-Doped TiO2 Nanobelt Array for High-Efficiency Electrocatalytic Nitrite Reduction to Ammonia. *Mater. Today Phys.* **2023**, *30*, 100944. https://doi.org/10.1016/j.mtphys.2022.100944.
(33) He, X.; Hu, L.; Xie, L.; Li, Z.; Chen, J.; Li, X.; Li, J.; Zhang, L.; Fang, X.; Zheng, D.; Sun, S.; Zhang, J.; Ali Alshehri, A.; Luo, Y.; Liu, Q.; Wang, Y.; Sun, X. Ambient Ammonia Synthesis via Nitrite Electroreduction over NiS2 Nanoparticles-Decorated TiO2 Nanoribbon Array. *J. Colloid Interface Sci.* **2023**, *634*, 86–92. https://doi.org/10.1016/j.jcis.2022.12.042.
(34) Zhao, D.; Liang, J.; Li, J.; Zhang, L.; Dong, K.; Yue, L.; Luo, Y.; Ren, Y.; Liu, Q.; Hamdy, M. S.; Li, Q.; Kong, Q.; Sun, X. A TiO2−x Nanobelt Array with Oxygen Vacancies: An Efficient Electrocatalyst toward Nitrite Conversion to Ammonia. *Chem. Commun.* **2022**, *58* (22), 3669–3672. https://doi.org/10.1039/D2CC00856D.
(35) Ouyang, L.; He, X.; Sun, S.; Luo, Y.; Zheng, D.; Chen, J.; Li, Y.; Lin, Y.; Liu, Q.; Asiri, A. M.; Sun, X. Enhanced Electrocatalytic Nitrite Reduction to Ammonia over P-Doped TiO2 Nanobelt Array. *J. Mater. Chem. A* **2022**, *10* (44), 23494–23498. https://doi.org/10.1039/D2TA06933D.
(36) He, X.; Li, X.; Fan, X.; Li, J.; Zhao, D.; Zhang, L.; Sun, S.; Luo, Y.; Zheng, D.; Xie, L.; Asiri, A. M.; Liu, Q.; Sun, X. Ambient Electroreduction of Nitrite to Ammonia over Ni Nanoparticle Supported on Molasses-Derived Carbon Sheets. *ACS Appl. Nano Mater.* **2022**, *5* (10), 14246–14250. https://doi.org/10.1021/acsanm.2c03720.
(37) Yang, X.; Kang, L.; Wang, C.-J.; Liu, F.; Chen, Y. Electrochemical Ammonia Synthesis from Nitrite Assisted by in Situ Generated Hydrogen Atoms on a Nickel Phosphide Catalyst. *Chem. Commun.* **2021**, *57* (58), 7176–7179. https://doi.org/10.1039/D1CC02410H.
(38) Yuan, X.; Chen, J.; Ling, Y.; Yu, S.; Li, S.; Wu, X.; Zhang, Z. A Facile and Efficient Nitrite Electrochemical Sensor Based on N, O Co-Doped Porous Graphene Film. *Microchem. J.* **2022**, *178*, 107361. https://doi.org/10.1016/j.microc.2022.107361.
(39) Zou, C.; Yang, B.; Bin, D.; Wang, J.; Li, S.; Yang, P.; Wang, C.; Shiraishi, Y.; Du, Y. Electrochemical Synthesis of Gold Nanoparticles Decorated Flower-like Graphene for High Sensitivity Detection of Nitrite. *J. Colloid Interface Sci.* **2017**, *488*, 135–141. https://doi.org/10.1016/j.jcis.2016.10.088.
(40) Rao, D.; Zhang, J.; Zheng, J. A Novel Electrochemical Sensor Based on Gold Nanorods and Nafion-Modified GCE for the Electrocatalytic Oxidation of Nitrite. *J. Iran. Chem. Soc.* **2016**, *13* (12), 2257–2266. https://doi.org/10.1007/s13738-016-0944-5.
(41) Govindasamy, M.; Wang, S.-F.; Huang, C.-H.; Alshgari, R. A.; Ouladsmane, M. Colloidal Synthesis of Perovskite-Type Lanthanum Aluminate Incorporated Graphene Oxide Composites: Electrochemical Detection of Nitrite in Meat Extract and Drinking Water. *Microchim. Acta* **2022**, *189* (5), 210. https://doi.org/10.1007/s00604-022-05296-4.
(42) Li, X.; Yang, X.; Cui, M.; Liu, Y.; Wang, J.; Zhang, L.; Zhan, G. A Novel Electrochemical Sensor Based on Nitrite-Oxidizing Bacteria for Highly Specific and Sensitive Detection of Nitrites. *Sci. Total Environ.* **2022**, *826*, 154178. https://doi.org/10.1016/j.scitotenv.2022.154178.

(43) Rashed, Md. A.; Faisal, M.; Harraz, F. A.; Jalalah, M.; Alsaiari, M.; Al-Assiri, M. S. rGO/ZnO/Nafion Nanocomposite as Highly Sensitive and Selective Amperometric Sensor for Detecting Nitrite Ions (NO2−). *J. Taiwan Inst. Chem. Eng.* **2020**, *112*, 345–356. https://doi.org/10.1016/j.jtice.2020.05.015.
(44) Wang, X.; Li, M.; Yang, S.; Shan, J. A Novel Electrochemical Sensor Based on TiO2–Ti3C2TX/CTAB/Chitosan Composite for the Detection of Nitrite. *Electrochimica Acta* **2020**, *359*, 136938. https://doi.org/10.1016/j.electacta.2020.136938.
(45) Zhe, T.; Li, R.; Li, F.; Liang, S.; Shi, D.; Sun, X.; Liu, Y.; Cao, Y.; Bu, T.; Wang, L. Surface Engineering of Carbon Selenide Nanofilms on Carbon Cloth: An Advanced and Ultrasensitive Self-Supporting Binder-Free Electrode for Nitrite Sensing. *Food Chem.* **2021**, *340*, 127953. https://doi.org/10.1016/j.foodchem.2020.127953.
(46) Zhe, T.; Li, M.; Li, F.; Li, R.; Bai, F.; Bu, T.; Jia, P.; Wang, L. Integrating Electrochemical Sensor Based on MoO3/Co3O4 Heterostructure for Highly Sensitive Sensing of Nitrite in Sausages and Water. *Food Chem.* **2022**, *367*, 130666. https://doi.org/10.1016/j.foodchem.2021.130666.
(47) Yang, Z.; Zhou, X.; Yin, Y.; Xue, H.; Fang, W. Metal-Organic Framework Derived Rod-like Co@carbon for Electrochemical Detection of Nitrite. *J. Alloys Compd.* **2022**, *911*, 164915. https://doi.org/10.1016/j.jallcom.2022.164915.
(48) Mariyappan, V.; Chen, S.-M.; Murugan, K.; Jeevika, A.; Jeyapragasam, T.; Ramachandran, R. Electrochemical Sensor Based on Cobalt Ruthenium Sulfide Nanoparticles Embedded on Boron Nitrogen Co-Doped Reduced Graphene Oxide for the Determination of Nitrite. *Colloids Surf. Physicochem. Eng. Asp.* **2022**, *637*, 128271. https://doi.org/10.1016/j.colsurfa.2022.128271.
(49) Talbi, M.; Al-Hamry, A.; Teixeira, P. R.; Paterno, L. G.; Ali, M. B.; Kanoun, O. Enhanced Nitrite Detection by a Carbon Screen Printed Electrode Modified with Photochemically-Made AuNPs. *Chemosensors* **2022**, *10* (2), 40. https://doi.org/10.3390/chemosensors10020040.
(50) Liu, J.-X.; Richards, D.; Singh, N.; Goldsmith, B. R. Activity and Selectivity Trends in Electrocatalytic Nitrate Reduction on Transition Metals. *ACS Catal.* **2019**, *9* (8), 7052–7064. https://doi.org/10.1021/acscatal.9b02179.
(51) Chun, H.-J.; Zeng, Z.; Greeley, J. DFT Insights into NO Electrochemical Reduction: A Case Study of Pt(211) and Cu(211) Surfaces. *ACS Catal.* **2022**, *12* (2), 1394–1402. https://doi.org/10.1021/acscatal.1c04493.
(52) Huo, X.; Van Hoomissen, D. J.; Liu, J.; Vyas, S.; Strathmann, T. J. Hydrogenation of Aqueous Nitrate and Nitrite with Ruthenium Catalysts. *Appl. Catal. B Environ.* **2017**, *211*, 188–198. https://doi.org/10.1016/j.apcatb.2017.04.045.
(53) Ren, Y.; Zhou, Q.; Li, J.; He, X.; Fan, X.; Fu, Y.; Fang, X.; Cai, Z.; Sun, S.; Hamdy, M. S.; Zhang, J.; Gong, F.; Liu, Y.; Sun, X. Ruthenium Doping: An Effective Strategy for Boosting Nitrite Electroreduction to Ammonia over Titanium Dioxide Nanoribbon Array. *J. Colloid Interface Sci.* **2023**, *645*, 806–812. https://doi.org/10.1016/j.jcis.2023.05.020.
(54) Troutman, J. P.; Li, H.; Haddix, A. M.; Kienzle, B. A.; Henkelman, G.; Humphrey, S. M.; Werth, C. J. PdAg Alloy Nanocatalysts: Toward Economically Viable Nitrite Reduction in Drinking Water. *ACS Catal.* **2020**, *10* (14), 7979–7989. https://doi.org/10.1021/acscatal.0c01538.
(55) Wang, Y.; Zhou, W.; Jia, R.; Yu, Y.; Zhang, B. Unveiling the Activity Origin of a Copper-Based Electrocatalyst for Selective Nitrate Reduction to Ammonia. *Angew. Chem. Int. Ed.* **2020**, *59* (13), 5350–5354. https://doi.org/10.1002/anie.201915992.
(56) Li, X.; Li, Z.; Zhang, L.; Zhao, D.; Li, J.; Sun, S.; Xie, L.; Liu, Q.; Ali Alshehri, A.; Luo, Y.; Liao, Y.; Kong, Q.; Sun, X. Ni Nanoparticle-Decorated Biomass Carbon for Efficient Electrocatalytic Nitrite Reduction to Ammonia. *Nanoscale* **2022**, *14* (36), 13073–13077. https://doi.org/10.1039/D2NR03540E.
(57) *Electrochemically Selective Ammonia Extraction from Nitrate by Coupling Electron- and Phase-Transfer Reactions at a Three-Phase Interface | Environmental Science & Technology*. https://pubs.acs.org/doi/full/10.1021/acs.est.0c08552 (accessed 2023-11-07).
